# Nanoemulsion based on natural components enables oral liver-targeting delivery of Schizandrol B to enhance liver regeneration after hepatectomy in hepatocellular carcinoma

**DOI:** 10.1093/rb/rbag019

**Published:** 2026-02-13

**Authors:** Yi Chen, Jinzhuan Xu, Jing Huang, Huizhou He, Zhimei Cheng, Zhengli Zhou, Man Zhang, Yuanxing Huang, Cao Huang, Jianqing Peng, Shuai Zhang, Runbin Sun, Zipeng Gong

**Affiliations:** State Key Laboratory of Discovery and Utilization of Functional Components in Traditional Chinese Medicine, School of Pharmaceutical Sciences, Guizhou Medical University, Guiyang 561113, China; Guizhou Provincial Key Laboratory of Innovation and Manufacturing for Pharmaceuticals, Guizhou Provincial Engineering Technology Research Center for Chemical Drug R&D, Guiyang 561113, China; State Key Laboratory of Discovery and Utilization of Functional Components in Traditional Chinese Medicine, School of Pharmaceutical Sciences, Guizhou Medical University, Guiyang 561113, China; State Key Laboratory of Discovery and Utilization of Functional Components in Traditional Chinese Medicine, School of Pharmaceutical Sciences, Guizhou Medical University, Guiyang 561113, China; State Key Laboratory of Discovery and Utilization of Functional Components in Traditional Chinese Medicine, School of Pharmaceutical Sciences, Guizhou Medical University, Guiyang 561113, China; Department of Interventional Radiology, The Affiliated Hospital of Guizhou Medical University, Guiyang 550004, China; State Key Laboratory of Discovery and Utilization of Functional Components in Traditional Chinese Medicine, School of Pharmaceutical Sciences, Guizhou Medical University, Guiyang 561113, China; Department of Interventional Radiology, The Affiliated Hospital of Guizhou Medical University, Guiyang 550004, China; State Key Laboratory of Discovery and Utilization of Functional Components in Traditional Chinese Medicine, School of Pharmaceutical Sciences, Guizhou Medical University, Guiyang 561113, China; State Key Laboratory of Discovery and Utilization of Functional Components in Traditional Chinese Medicine, School of Pharmaceutical Sciences, Guizhou Medical University, Guiyang 561113, China; State Key Laboratory of Discovery and Utilization of Functional Components in Traditional Chinese Medicine, School of Pharmaceutical Sciences, Guizhou Medical University, Guiyang 561113, China; State Key Laboratory of Discovery and Utilization of Functional Components in Traditional Chinese Medicine, School of Pharmaceutical Sciences, Guizhou Medical University, Guiyang 561113, China; State Key Laboratory of Discovery and Utilization of Functional Components in Traditional Chinese Medicine, School of Pharmaceutical Sciences, Guizhou Medical University, Guiyang 561113, China; Guizhou Provincial Key Laboratory of Innovation and Manufacturing for Pharmaceuticals, Guizhou Provincial Engineering Technology Research Center for Chemical Drug R&D, Guiyang 561113, China; State Key Laboratory of Discovery and Utilization of Functional Components in Traditional Chinese Medicine, School of Pharmaceutical Sciences, Guizhou Medical University, Guiyang 561113, China; Department of Interventional Radiology, The Affiliated Hospital of Guizhou Medical University, Guiyang 550004, China; Phase I Clinical Trials Unit, Nanjing Drum Tower Hospital, Affiliated Hospital of Medical School, Nanjing University, Nanjing 210008, China; State Key Laboratory of Discovery and Utilization of Functional Components in Traditional Chinese Medicine, School of Pharmaceutical Sciences, Guizhou Medical University, Guiyang 561113, China

**Keywords:** nanoemulsion, Schizandrol B, liver regeneration, partial hepatectomy, hepatocellular carcinoma

## Abstract

Hepatocellular carcinoma (HCC) remains a major cause of cancer-related mortality worldwide, with partial hepatectomy (PHx) serving as the primary curative treatment. Schizandrol B (SCHB) has demonstrated significant efficacy in promoting liver regeneration and restoring hepatic function following PHx. However, the clinical application of SCHB faces two critical challenges: poor oral bioavailability and inadequate liver-specific targeting. Here, this study developed a nanoemulsion based on natural components named SCHB@SPC/Gal-BSA/DHA. It effectively overcame the limitations of SCHB by synergizing Gal-BSA-mediated liver-specific targeting and DHA-enhanced intestinal absorption, achieving prolonged gastrointestinal tract retention, 2.03-fold higher oral bioavailability and 7.64-fold greater liver accumulation compared to free SCHB. In both 70% PHx and *in situ* PHx in HCC models, the nanoemulsion robustly accelerated liver regeneration, evidenced by upregulated proliferation markers *via* STAT3/YAP activation and normalized bile acids metabolism, ultimately restoring liver mass faster than control. This study demonstrates that the dual-targeted nanoemulsion effectively overcomes the key limitations of SCHB by combining enhanced intestinal absorption with liver-specific targeting. The developed nanoemulsion system not only improves drug delivery efficiency but also significantly promotes liver regeneration after PHx, offering a promising therapeutic approach for postoperative recovery in HCC patients while establishing a platform for future liver-targeted oral drug delivery systems.

## Graphical Abstract

**Graphical Abstract:**
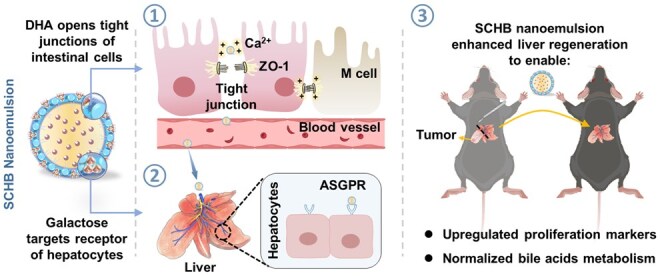

## Introduction

Hepatocellular carcinoma (HCC) constitutes about 80% of primary liver cancers and is the third leading cause of cancer-related deaths globally [1, 2]. Partial hepatectomy (PHx) remains the primary curative treatment, but the postoperative hepatic microenvironment, especially in diseased livers, significantly impairs the innate regenerative capacity of the liver [3]. Although the normal liver can fully regenerate even after 70% PHx [4], this process is critically undermined by tumor-associated pathology. The resulting failure of residual tissue regeneration directly threatens patient survival, highlighting the urgent need for safe and effective therapies that can robustly promote liver regeneration [5].

The current strategies for promoting liver regeneration are facing numerous challenges. The regeneration of post-operative liver tissue requires both cell quantity restoration and metabolic function stability [e.g. bile acid (BA) metabolism and detoxification]. Recent studies have confirmed that metabolic reprogramming is not merely an accompanying phenomenon, but rather the core engine that initiates and coordinates the regeneration program. Metabolic imbalance can hinder the regeneration process, exacerbate injury and compromise surgical outcomes. Therefore, elucidating how metabolism drives proliferation and ultimately serves functional recovery lays a critical theoretical foundation for understanding subsequent drug actions. Conventional pharmacological agents often struggle to simultaneously drive hepatocyte proliferation and maintain metabolic homeostasis, with some raising concerns over potential tumorigenic risks from prolonged pathway activation [6–10]. Biological therapies, such as stem cells or growth factors, are promising but face challenges including immune rejection and uncertain long-term efficacy [11, 12]. Additionally, the clinical applicability of microencapsulated human hepatocyte organoids for treating massive hepatectomy-induced liver failure awaits further validation [13, 14]. Given these limitations, extracts or monomers from traditional Chinese medicine (TCM) have garnered increasing attention as a valuable resource for postoperative recovery, owing to their established safety profile and multitargeted effects [9, 15].

Among these TCM-derived compounds, Schizandrol B (SCHB), a bioactive lignan from Schisandra chinensis, has demonstrated significant efficacy in promoting liver regeneration and restoring function by activating pivotal pathways like PXR-YAP [16–18]. Furthermore, SCHB reduces inflammation during regeneration, thereby normalizing serum liver biomarkers and maintaining functional integrity [19–21]. However, clinical transformation of SCHB is severely limited by inherent pharmacokinetic deficiencies, particularly poor solubility, low oral bioavailability and insufficient hepatic accumulation, with few dedicated oral delivery systems [22, 23]. These deficiencies primarily arise from its high lipophilicity, rigid fused-ring structure and stable crystal packing, which collectively result in poor aqueous solubility and consequently low effective concentration in the gastrointestinal tract, limiting oral absorption. Moreover, SCHB is mainly catalyzed by hepatic CYP3A enzymes, which greatly reduces the proportion of parent drug entering the systemic circulation through phase I reactions (e.g. demethylation, hydroxylation). After phase II metabolism, metabolites of SCHB are mainly excreted through bile and feces, further reducing the systemic availability of parent compound [24]. Consequently, an advanced oral drug delivery system is urgently required to enhance the solubility, intestinal absorption and liver-specific targeting of SCHB.

Various drug delivery systems, including emulsions [25], liposomes [26], cyclodextrins [27], solid lipid nanoparticles [28], polymeric nanoparticles [29] and probiotics [30] have been used to address the challenges associated with poor solubility and targeted drug delivery. We, therefore, focused on a phospholipid-based nanoemulsion, leveraging its superior biocompatibility, proven industrial viability and the precedent of approved phospholipid emulsions [31]. Our formulation integrates two functionally distinct targeting strategies to overcome the sequential barriers of oral drug delivery. First, to overcome the intestinal absorption barrier, we incorporated docosahexaenoic acid (DHA), which reversibly loosens tight junctions (TJs) by modulating proteins such as Zonula occludens-1 (ZO-1), thereby facilitating paracellular transport of nanocarriers [32, 33]. Our preliminary data corroborate this mechanism, confirming DHA’s role as an effective absorption enhancer. Second, to actively direct the absorbed nanoemulsion to the liver, we employed glycosylated bovine serum albumin (Gal-BSA) as a co-emulsifier. Synthesized *via* a controlled Maillard reaction between BSA and D-N-acetylgalactosamine (Gal), this conjugate exploits the high-affinity binding of galactose moieties to asialoglycoprotein receptors (ASGPR), which are abundantly expressed on hepatocytes [34–36].

**Figure 1:**
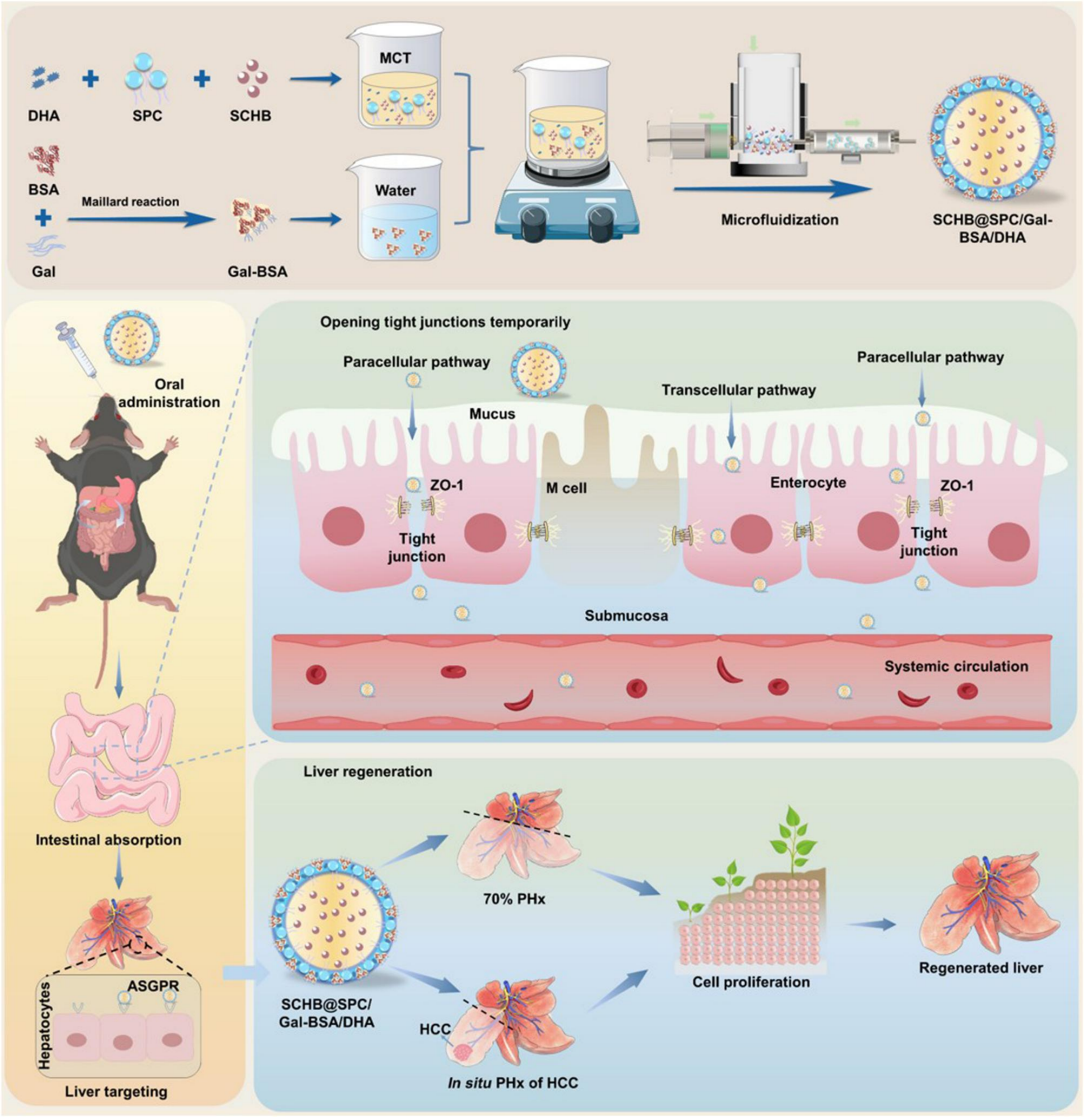
SCHB nanoemulsions promote oral absorption and liver regeneration.

In conclusion, we developed an innovative oral nanoemulsion, SCHB@SPC/Gal-BSA/DHA, which synergistically integrates soybean phospholipid (SPC)-based encapsulation, DHA-enhanced intestinal permeability and Gal-BSA-mediated active liver targeting (Figure 1). Composed of natural components with high biocompatibility, this system is engineered to efficiently navigate the journey from oral administration to hepatic accumulation. Beyond conventional pharmacokinetic and biodistribution studies, we comprehensively evaluated this formulation, demonstrating its superior pharmacokinetics and, critically, its potent efficacy in promoting functional liver regeneration in both healthy and HCC-bearing mice after PHx, with a particular focus on functional liver regeneration markers and normalization of BA metabolism. This work, thus, establishes a new liver regeneration paradigm by solving the key delivery challenges of SCHB, offering a promising strategy to improve outcomes for surgical liver cancer patients.

The Experimental section is presented in the Supplementary Materials.

## Results

### Optimization and preparation of SCHB@SPC/Gal-BSA/DHA

A series of Gal-BSA galactosylated conjugates was synthesized under dry heating conditions by systematically optimizing the reaction pH, reaction time and protein-to-reducing sugar ratio. Qualitative analysis by sodium dodecyl sulfate-polyacrylamide gel electrophoresis (SDS-PAGE) showed that BSA exhibited a distinct band at 63–75 kDa, while a smeared band appeared at >75 kDa after the Maillard reaction, confirming the successful formation of Gal-BSA conjugates (Supplementary Figure S1A, D and G). The degree of glycosylation (DG) was quantitatively evaluated using the Ortho-Phthalaldehyde method (Supplementary Figure S1B, E and H). Additionally, absorbance measurements at 294 nm and 420 nm were employed to monitor the formation of melanoidins and the concentration of glycosylated proteins, respectively (Supplementary Figure S1C, F and I).

**Figure 2:**
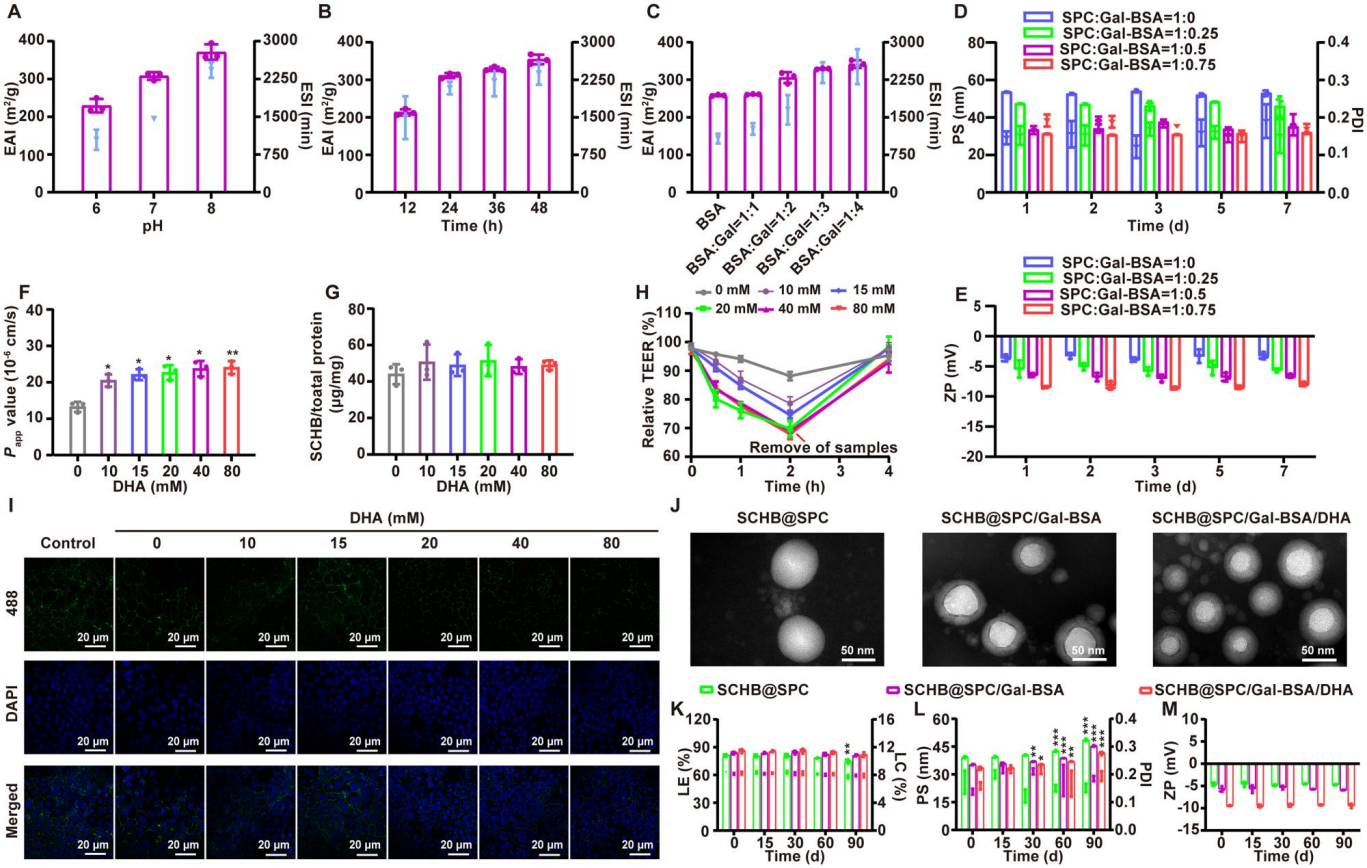
Optimization and preparation of SCHB@SPC/Gal-BSA/DHA. EAI and ESI of Gal-BSA at various (**A**) pH values, (**B**) reaction times and (**C**) ratios. Changes in (**D**) PS, PDI and (**E**) ZP of SCHB@SPC/Gal-BSA at different ratios at 4°C for 7 days. (**F**) *P*_app_, (**G**) uptake and (**H**) TEER of SCHB@SPC/Gal-BSA/DHA at different DHA contents. *P < 0.05 and **P < 0.01 compared with 0 mM. (**I**) ZO-1 staining of SCHB@SPC/Gal-BSA/DHA at different DHA contents. Scale bar, 20 μm. (**J**) TEM images of SCHB@SPC, SCHB@SPC/Gal-BSA and SCHB@SPC/Gal-BSA/DHA. Scale bar, 50 nm. Changes in (**K**) LE and loading capacity (LC), (**L**) PS and PDI and (**M**) ZP of SCHB nanoemulsions stored at 4°C for 90 days. *P < 0.05, **P < 0.01 and ***P < 0.001 compared with 0 day. All data are presented as the mean ± standard deviation (SD; *n *= 3).

The emulsifying capacity of Gal-BSA, assessed *via* the emulsification activity index (EAI) and emulsion stability index (ESI), was found to correlate positively with the DG value (Figure 2A–C). Based on these results, the reaction conditions were optimized to pH 8, 24 h and a BSA-to-Gal mass ratio of 1:3 to obtain Gal-BSA with high DG and enhanced emulsifying properties. When used as a co-emulsifier with SPC, increasing the proportion of Gal-BSA led to the formation of smaller and more stable nanoemulsions (Figure 2D and E). The optimal mass ratio of SPC to Gal-BSA was determined to be 1:0.5.

To enhance intestinal absorption, DHA was incorporated into the nanoemulsion. The apparent permeability coefficient (*P*_app_) of SCHB across Caco-2 cell monolayers increased with rising DHA content (Figure 2F). Cellular uptake was not affected by DHA content (Figure 2G), whereas the transepithelial electrical resistance (TEER) was reversibly reduced (Figure 2H). Immunofluorescence staining of ZO-1 further indicated that DHA facilitated the transient opening of TJs, likely through complexation with extracellular Ca^2+^ which affects ZO-1 function (Figure 2I and Supplementary Figure S2). Based on these findings, a DHA content of 20 mM was selected for subsequent formulations, and SCHB@SPC/Gal-BSA/DHA was prepared at an SPC-to-Gal-BSA ratio of 1:0.5.

Transmission electron microscopy (TEM) revealed that all nanoemulsions were spherical, well-dispersed and uniform in morphology (Figure 2J). SCHB@SPC/Gal-BSA and SCHB@SPC/Gal-BSA/DHA displayed a hazy hydrated layer on the surface, attributable to the incorporation of Gal-BSA. Storage stability was evaluated at 4°C over 90 days. Loading efficiency (LE) remained above 80% for all formulations (Figure 2K). SCHB@SPC showed a rapid increase in particle size (PS) and a rise in polydispersity index (PDI) from 0.1 to around 0.2. In contrast, SCHB@SPC/Gal-BSA and SCHB@SPC/Gal-BSA/DHA exhibited slower growth in PS and narrower size distribution (Figure 2L). No notable changes in zeta potential (ZP) were observed in any group during storage (Figure 2M). In addition, the stability of the product under more severe conditions was investigated with reference to the stability technical guidelines, and the results (Supplementary Figure S3) demonstrate the excellent stability of the nanoemulsion.

### Absorption mechanisms of SCHB nanoemulsions

**Figure 3:**
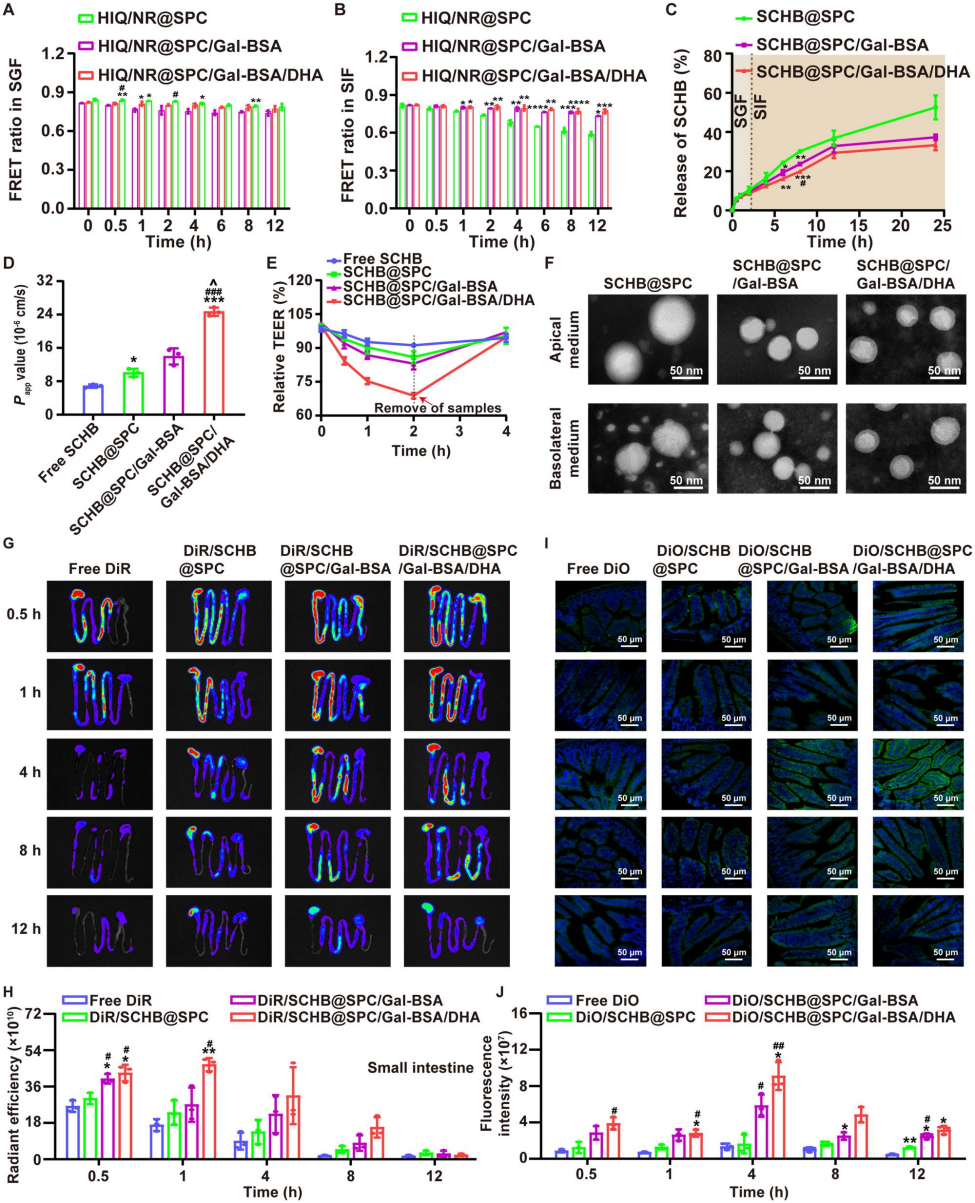
FRET intensity of nanoemulsions prepared with HIQ and NR after incubation in (**A**) SGF and (**B**) SIF at different time points. *P < 0.05, **P < 0.01, ***P < 0.001 and ****P < 0.0001 compared with HIQ/NR@SPC; ^#^*P *< 0.05 compared with HIQ/NR@SPC/Gal-BSA. (**C**) Drug release profile of SCHB from SCHB@SPC, SCHB@SPC/Gal-BSA and SCHB@SPC/Gal-BSA/DHA in SGF and SIF. *P < 0.05, **P < 0.01 and ***P < 0.001 compared with SCHB@SPC; ^#^*P *< 0.05 compared with SCHB@SPC/Gal-BSA. (**D**) *P*_app_ values for the SCHB nanoemulsions transported across the Caco-2 cell monolayer after 2 h. (**E**) Relative changes in TEER in the Caco-2 cell monolayer after treatment with SCHB nanoemulsions. *P < 0.05 and ***P < 0.001 compared with free SCHB; ^###^*P *< 0.001 compared with SCHB@SPC; ^^^*P *< 0.05 compared with SCHB@SPC/Gal-BSA. (**F**) TEM images of SCHB@SPC, SCHB@SPC/Gal-BSA and SCHB@SPC/Gal-BSA/DHA at the apical and basolateral sides of the Caco-2 cell monolayer after incubation in MEM at 37°C for 2 h. Scale bar, 50 nm. (**G**) Fluorescent signals and (**H**) quantification of free DiR, DiR/SCHB@SPC, DiR/SCHB@SPC/Gal-BSA and DiR/SCHB@SPC/Gal-BSA/DHA in the small intestine at various time points after oral administration. *P < 0.05 and **P < 0.01 compared with free DiR; ^#^*P *< 0.05 compared with DiR/SCHB@SPC. (**I**) Representative fluorescence images and (**J**) quantification of ileal segments after oral administration of DiO nanoemulsions. DiO is shown in green and the nuclei are shown in blue (stained with DAPI). Scale bar, 50 μm. *P < 0.05 and **P < 0.01 compared with free DiO; ^#^*P *< 0.05 and ^##^*P *< 0.01 compared with DiO/SCHB@SPC. All data are presented as the mean ± SD (*n *= 3).

The stability of the nanoemulsions in the gastrointestinal tract was evaluated using a FRET-based method [37]. In simulated gastric fluid (SGF), the FRET ratio (FR) for all three nanoemulsions decreased slowly over time (Figure 3A). In simulated intestinal fluid (SIF), the FR of HIQ/NR@SPC declined rapidly after 2 h, whereas the FR of HIQ/NR@SPC/Gal-BSA and HIQ/NR@SPC/Gal-BSA/DHA remained largely unchanged (Figure 3B). The superior stability of HIQ/NR@SPC/Gal-BSA and HIQ/NR@SPC/Gal-BSA/DHA in SIF is attributed to the hydrophilic barrier formed by Gal-BSA, which hinders the access and binding of pancreatic lipase. The intrinsic solubility of SCHB was 0.79 mg/mL in SGF and 0.73 mg/mL in SIF. SCHB@SPC/Gal-BSA/DHA enhanced the solubility of SCHB to levels 13.46- and 14.56-fold higher in SGF and SIF, respectively (Supplementary Figure S4). During *in vitro* release studies, SCHB@SPC showed a cumulative release of 52.60% over 24 h. In contrast, SCHB@SPC/Gal-BSA and SCHB@SPC/Gal-BSA/DHA exhibited lower cumulative release rates of 37.32% and 33.33%, respectively (Figure 3C).

The permeability of SCHB nanoemulsions was assessed using Caco-2 cell monolayers. Both blank and drug-loaded nanoemulsions showed no significant cytotoxicity, with cell viability exceeding 90% after 24 h of incubation (Supplementary Figure S5). The P_ap__*p*_ values of the SCHB nanoemulsions were approximately 2- to 3-fold higher than those of free SCHB, with SCHB@SPC/Gal-BSA/DHA showing the greatest enhancement (Figure 3D). All nanoemulsions induced a rapid decrease in TEER, with the SCHB@SPC/Gal-BSA/DHA group showing a 68.83% reduction at 2 h; TEER recovered after nanoemulsion removal (Figure 3E). ZO-1 staining revealed alterations in TJs morphology following nanoemulsion treatment (Supplementary Figure S6). TEM images of the Caco-2 monolayer after incubation showed that SCHB@SPC exhibited irregular, unrounded droplets on the basolateral side, indicating nanoemulsion destabilization (Figure 3F).

*In vivo* gastrointestinal tracking after oral administration showed that nanoemulsions were retained in the stomach longer than free DiR (Figure 3G). At 4 h, fluorescence in the small intestine was weak for free DiR but remained detectable in all nanoemulsion groups, with DiR/SCHB@SPC/Gal-BSA and DiR/SCHB@SPC/Gal-BSA/DHA showing the strongest retention (Figure 3H and Supplementary Figure S7). In ileal tissue sections, free DiO produced weak fluorescence, whereas DiO-labeled nanoemulsions were clearly visible in the brush border region of intestinal villi (Figure 3I). Quantitative analysis confirmed that all nanoemulsions, particularly DiO/SCHB@SPC/Gal-BSA/DHA, led to prolonged intestinal fluorescence retention (Figure 3J).

### Liver accumulation of SCHB nanoemulsions after oral administration

**Figure 4:**
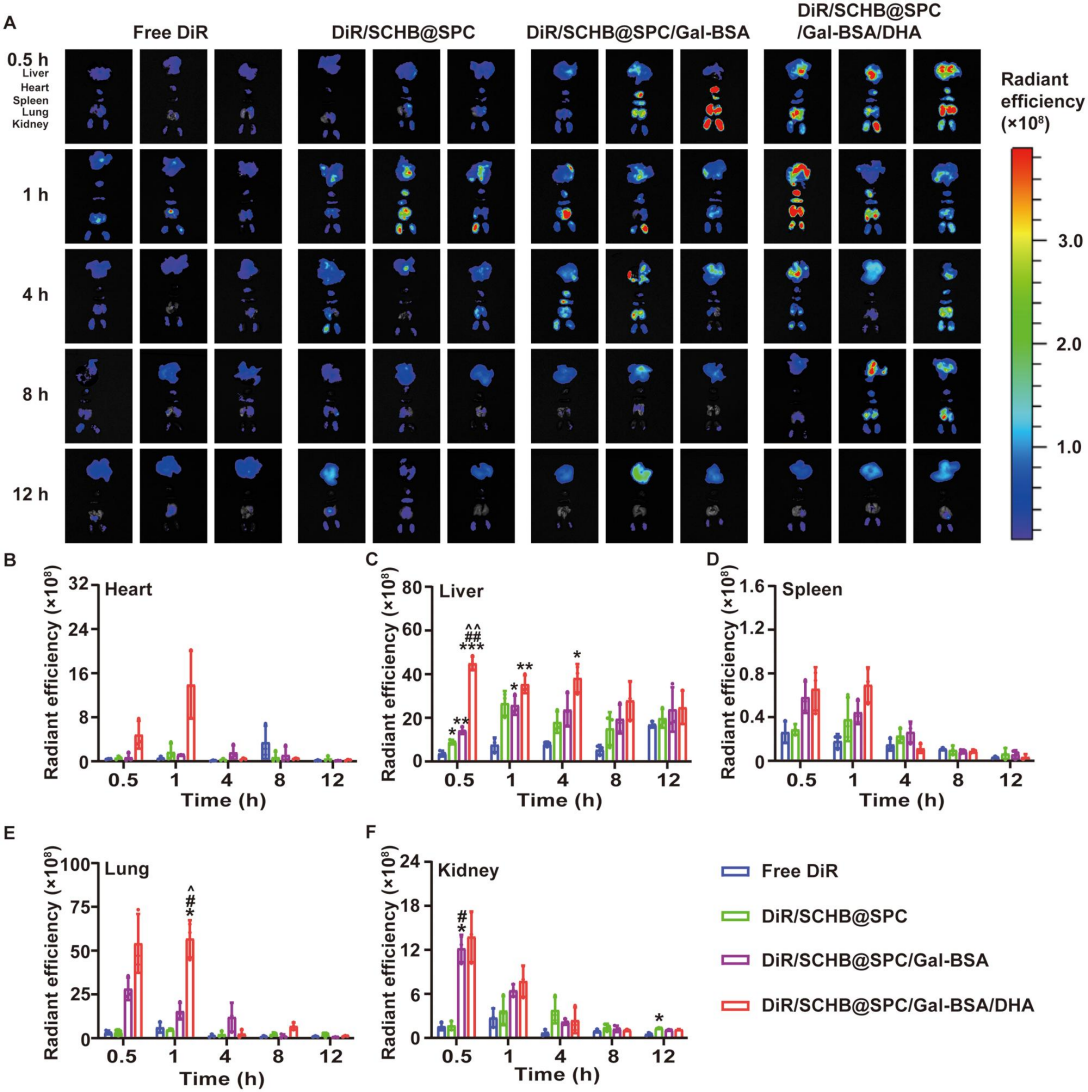
Liver accumulation of SCHB nanoemulsions after oral administration. (**A**) Accumulation of nanoemulsions in tissues as imaged by IVIS 12 h after the oral administration of DiR nanoemulsions (2 mg/kg). Quantitation of radiant efficiency for (**B**) heart, (**C**) liver, (**D**) spleen, (**E**) lung and (**F**) kidney. *P < 0.05, **P < 0.01 and ***P < 0.001 compared with free DiR; ^#^*P *< 0.05 and ^##^*P *< 0.01 compared with DiR/SCHB@SPC; ^^^*P *< 0.05 and ^^^^*P *< 0.01 compared with DiR/SCHB@SPC/Gal-BSA. All data are presented as the mean ± SD (*n *= 3).

Free DiR, DiR/SCHB@SPC, DiR/SCHB@SPC/Gal-BSA or DiR/SCHB@SPC/Gal-BSA/DHA were orally administered to C57BL/6 mice, and their fluorescence intensities were monitored using an *in vivo* imaging spectrum system (IVIS) (Supplementary Figure S8). Tissue collection at different time points after drug administration for *ex vivo* imaging (Figure 4A). Semiquantitative analysis of DiR distribution across tissues showed that the free DiR group exhibited minimal fluorescence signals in all examined tissues (Figure 4B–F). In contrast, the DiR/SCHB@SPC/Gal-BSA/DHA group displayed the strongest fluorescence intensity in liver tissue at all time points. The enhanced liver accumulation is likely due to the synergistic effect of DHA, which improves intestinal absorption and systemic availability, and Gal-BSA, which acts as a ligand for the ASGPR on hepatocytes, facilitating active targeting to the liver.

### Pharmacokinetic properties and tissue distribution of SCHB nanoemulsions

**Figure 5:**
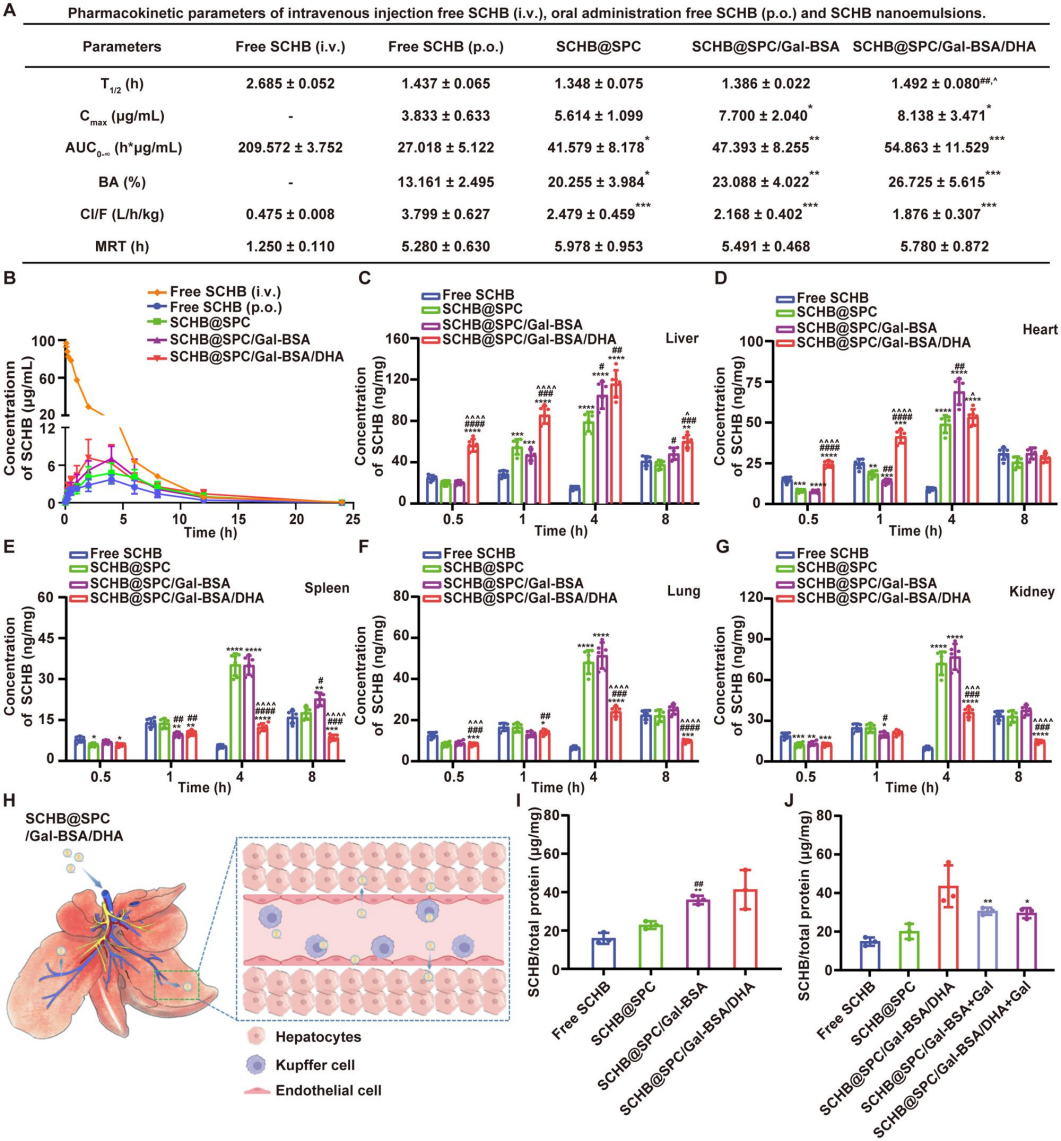
Pharmacokinetic properties and tissue distribution of SCHB nanoemulsions. (**B**) Drug concentration-time curves and (**A**) pharmacokinetic parameters of free SCHB (i.v.), free SCHB (p.o.), SCHB@SPC, SCHB@SPC/Gal-BSA and SCHB@SPC/Gal-BSA/DHA after 100 mg/kg treatment in mice. The distribution of SCHB in the (**C**) liver, (**D**) heart, (**E**) spleen, (**F**) lung and (**G**) kidney of mice after oral administration of free SCHB and SCHB nanoemulsions at a dose of 100 mg/kg. All data are presented as the mean ± SD (*n *= 6). (**H**) Schematic of the intercellular transport of SCHB@SPC/Gal-BSA/DHA in the liver. SCHB uptake in (**I**) AML12 cells after treatment with different SCHB nanoemulsions (*n *= 3). (**J**) SCHB uptake in AML12 cells preincubated with Gal (*n *= 3). *P < 0.05, **P < 0.01, ***P < 0.001 and ****P < 0.0001 compared with free SCHB; ^#^*P *< 0.05, ^##^*P *< 0.01, ^###^*P *< 0.001 and ^####^*P *< 0.0001 compared with SCHB@SPC; ^^^*P *< 0.05, ^^^^*P *< 0.01, ^^^^^*P *< 0.001 and ^^^^^^*P *< 0.0001 compared with SCHB@SPC/Gal-BSA.

The blood drug concentration-time profiles of C57BL/6 mice following the intravenous injection of free SCHB (i.v.) and oral administration of free SCHB (p.o.) or various SCHB nanoemulsions are shown in Figure 5B. Pharmacokinetic parameters derived from the noncompartmental analysis are summarized in Figure 5A. The area under the curve (AUC_0∼∞_) of SCHB for the SCHB@SPC/Gal-BSA and SCHB@SPC/Gal-BSA/DHA groups was significantly higher than that of the free SCHB (p.o.) group. The absolute bioavailability values of SCHB@SPC, SCHB@SPC/Gal-BSA and SCHB@SPC/Gal-BSA/DHA were 1.54-fold (95% CI: 15.89–24.08; *P *< 0.01), 1.76-fold (95% CI: 18.58-26.88; *P *< 0.001) and 2.03-fold (95% CI: 21.13–31.52; *P *< 0.0001), respectively, higher than that of free SCHB (p.o. 95% CI: 10.68–15.18), Supplementary Table S1.

Tissue distribution of SCHB was evaluated at 0.5, 1, 4 and 8 h after administration. SCHB@SPC/Gal-BSA/DHA showed rapid accumulation in the liver at 0.5 h (Figure 5C), with a maximum drug concentration of 115.83 ng/mg, which was 7.64-fold higher than that of the free SCHB group. The AUC of liver accumulation of SCHB@SPC, SCHB@SPC/Gal-BSA and SCHB@SPC/Gal-BSA/DHA were 2.31-fold (95% CI: 4001.74–5191.55; *P *< 0.0001), 2.80-fold (95% CI: 4856.3–6285.74; *P *< 0.0001) and 3.55-fold (95% CI: 6250.65–7852.07; *P *< 0.0001), respectively, higher than that of free SCHB (p.o. 95% CI: 1744.07–2229.36), Supplementary Table S1. SCHB@SPC and SCHB@SPC/Gal-BSA reached peak concentrations in non-liver tissues within 4 h, with no significant differences among these groups (Figure 5D–G). The highest SCHB concentrations were observed in the liver and kidneys.

A schematic of the liver microstructure illustrates potential cellular interactions of the nanoemulsion (Figure 5H). Uptake studies in AML12 cells showed that glycosylated nanoemulsions increased SCHB absorption (Figure 5I) within non-cytotoxic concentration ranges (Supplementary Figure S9). When AML12 cells were pre-saturated with Gal, the uptake of SCHB@SPC/Gal-BSA and SCHB@SPC/Gal-BSA/DHA was markedly reduced, while the uptake of free SCHB and SCHB@SPC remained unchanged. This competition assay confirms that the uptake of glycosylated nanoemulsions is mediated by the ASGPR (Figure 5J).

To gain a deeper insight into the impact of formulation design on efficacy, we analyzed the correlations of liver uptake, as well as serum and liver AUC, with the key physicochemical parameters of the nanoemulsion (e.g. PDI, PS and ZP) and liver regeneration-related indices (e.g. liver/body weight ratio). As shown in Supplementary Figures S10–S13, we observed a significant negative correlation of liver uptake and serum/liver AUC with ZP, while no significant correlations were found with the nanoemulsion’s PDI and PS. This suggests that a lower ZP may be more conducive to hepatic-targeted uptake. More importantly, a significant positive correlation was observed between the liver uptake, serum and liver AUC with liver regeneration efficiency, which strongly demonstrates, from a pharmacokinetic perspective, that our designed SCHB nanoemulsion drives the liver regeneration process by enhancing intrahepatic drug exposure levels.

### SCHB nanoemulsion promoted liver regeneration after 70% PHx

**Figure 6:**
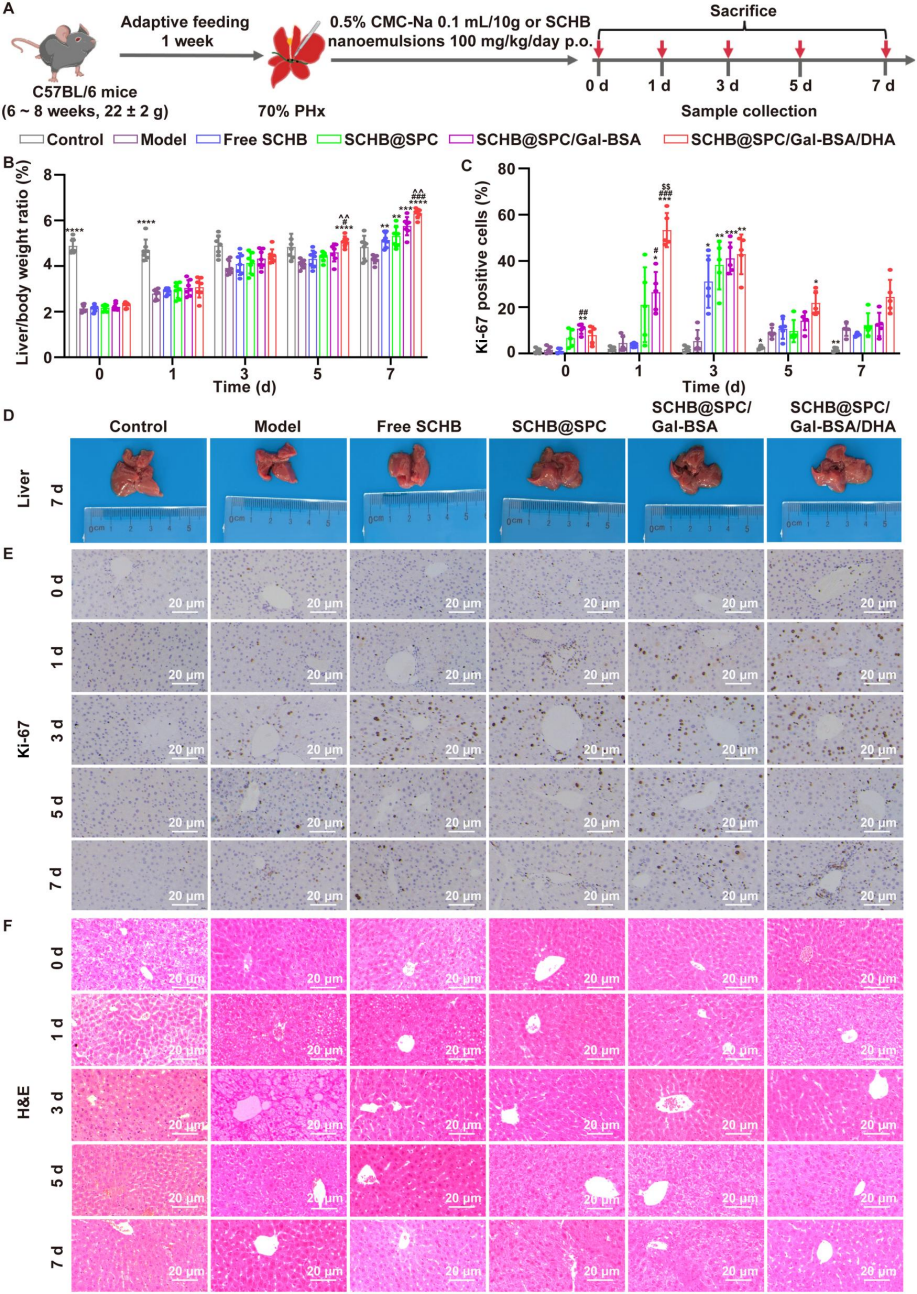
SCHB nanoemulsions promoted liver regeneration after 70% PHx. (**A**) Schematic of the animal treatment. (**B**) Liver/body weight ratios of mice (*n *= 7). (**D**) Liver on Day 7 after 70% PHx in the different groups. Liver sections were stained with (**C** and **E**) Ki-67 and (**F**) H&E. Ki-67-positive cells in at least five microscope fields per sample (*n *= 5). Scale bar, 20 μm. *P < 0.05, **P < 0.01, ***P < 0.001 and ****P < 0.0001 compared with the model; ^#^*P *< 0.05, ^##^*P *< 0.01 and ^###^*P *< 0.001 compared with free SCHB; ^^^^*P *< 0.01 compared with SCHB@SPC; and ^$$^*P *< 0.01 compared with SCHB@SPC/Gal-BSA. All data are presented as the mean ± SD.

To evaluate the effect of SCHB-loaded nanoemulsions on liver regeneration, mouse models of 70% PHx were established (Supplementary Figure S14). Mice were orally administered 100 mg/kg SCHB nanoemulsions daily, while control and model groups received 0.5% CMC-Na solution (Figure 6A). Liver/body weight ratios were measured at designated time points post-PHx (Figure 6B). The model group showed progressive liver enlargement over time. Morphological examination further confirmed an increase in liver volume after treatment (Figure 6D). On Day 7, the free SCHB and SCHB nanoemulsion groups exhibited significantly higher liver/body weight ratios than the model group. The ratio increased from 144.70% to 176.32% in SCHB nanoemulsion-treated groups relative to Day 0. The SCHB@SPC/Gal-BSA/DHA group showed the most pronounced increase, with a 46.51% higher ratio than the model group on Day 7. Routine blood tests (Supplementary Figure S15) and histopathological analysis (Supplementary Figure S16) revealed no significant changes in hematological parameters or tissue morphology, indicating no adverse effects from the nanoemulsions.

Hepatic cell proliferation was assessed by Ki-67 staining (Figure 6C and E). The nanoemulsion groups showed increased Ki-67-positive cells from Day 0 (2 h) onward, with the SCHB@SPC/Gal-BSA/DHA group having the highest proportion. By Day 1, over half of the cells in the SCHB@SPC/Gal-BSA/DHA group were Ki-67-positive. By Day 3, the other SCHB nanoemulsion groups reached similar Ki-67-positive levels. The free SCHB group showed a moderate increase in Ki-67-positive cells compared to the model group, though the difference was not statistically significant. Hematoxylin and eosin (H&E) staining (Figure 6F) revealed disordered cell arrangement and increased nuclear division in the model group. In contrast, livers from free SCHB and SCHB nanoemulsion-treated groups displayed clear cellular structure, orderly arrangement, uniform cell size and no abnormal nuclear division.

**Figure 7:**
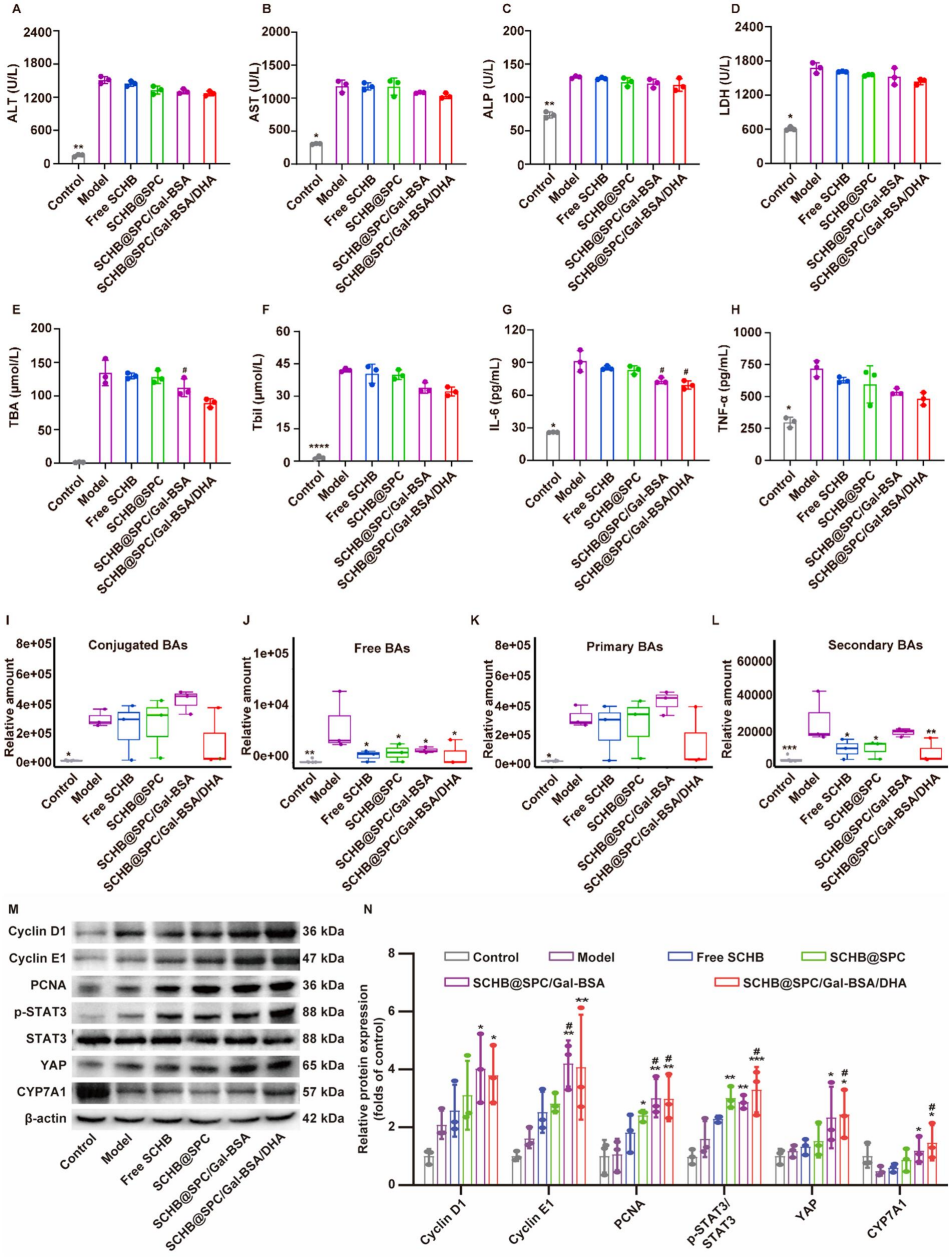
SCHB nanoemulsions promoted liver regeneration with 70% PHx by promoting STAT3 phosphorylation and YAP activation. Serum levels of (**A**) ALT, (**B**) AST, (**C**) ALP, (**D**) LDH, (**E**) TBA, (**F**) Tbil, (**G**) IL-6 and (**H**) TNF-α on Day 1. Relative amounts of (**I**) conjugated BAs, (**J**) free BAs, (**K**) primary BAs and (**L**) secondary BAs in the serum on Day 1 after 70% PHx. (**M** and **N**) Western blot analysis of Cyclin D1, Cyclin E1, PCNA, p-STAT3/STAT3, YAP and CYP7A1 on Day 1 after 70% PHx. *P < 0.05, **P < 0.01, ***P < 0.001 and ****P < 0.0001 compared with the model; and ^#^*P *< 0.05 compared with free SCHB. All data are presented as the mean ± SD (*n *= 3).

In murine models of 70% PHx, serum levels of alanine transaminase (ALT) and aspartate aminotransferase (AST) increased within 2 h post-surgery (Day 0) and gradually declined thereafter (Figure 7A and B and Supplementary Figure S17A and B). Free SCHB and SCHB nanoemulsions significantly reduced AST and ALT levels from postoperative Day 3. Similarly, elevations in alkaline phosphatase (ALP) and lactate dehydrogenase (LDH) induced by PHx were reversed by SCHB treatments, with the most notable effects on Day 7 (Figure 7C and D and Supplementary Figure S17C and D). Serum total bile acid (TBA) and total bilirubin (Tbil) levels were also significantly increased from 2 h after surgery (Figure 7E and F and Supplementary Figure S17E and F). Treatment with free SCHB and SCHB nanoemulsions markedly lowered TBA and Tbil levels compared to the model group, with nanoemulsions showing greater reduction than free SCHB. Levels of tumor necrosis factor-α (TNF-α) and interleukin-6 (IL-6) rose rapidly after PHx and gradually decreased during liver recovery (Figure 7G and H and Supplementary Figure S17G and H). Free SCHB and SCHB nanoemulsions accelerated the decline of TNF-α and IL-6, bringing them closer to control levels.

Serum levels of 15 BAs components were quantified on Day 1 post-PHx. Multiple BAs, including conjugated, free, primary and secondary types, were significantly elevated within 24 h (Figure 7I–L). SCHB administration generally reduced serum levels of free BAs (e.g. alphaMCA and betaMCA) and secondary BAs (e.g. omegaMCA and UDCA), whereas conjugated and primary BAs showed no obvious changes (Supplementary Figure S18). SCHB nanoemulsions, particularly SCHB@SPC/Gal-BSA/DHA, led to more pronounced reductions in BA levels than free SCHB.

Western blot analysis was performed to examine protein expression related to liver regeneration (Figure 7M and N and Supplementary Figure S19). The results showed that in mice administered free SCHB, all the genes tested showed a trend of increase compared with the Model group, but the differences were not significant. After treatment with SCHB@SPC, the expression of PCNA and p-STAT3/STAT3 was increased statistically significantly by 2.27-fold (*P *< 0.05) and 1.49-fold (*P *< 0.01), respectively. The changes in Cyclin D1, Cyclin E1, YAP and CYP7A1 did not reach statistical significance (1.49-fold, 1.74-fold, 1.29-fold, 1.80-fold, respectively; *P *> 0.05). SCHB@SPC/Gal-BSA increased the expression of Cyclin D1 (1.93-fold, *P *< 0.05), Cyclin E1 (2.61-fold, *P *< 0.01), PCNA (2.83-fold, *P *< 0.01), p-STAT3 (2.23-fold, *P *< 0.01), YAP (1.98-fold, *P *< 0.05) and CYP7A1 (2.46-fold, *P *< 0.05) significantly. In the SCHB@SPC/Gal-BSA/DHA group, the upregulation of gene expression was the most significant, Cyclin D1, Cyclin E1 and PCNA increased by 1.82-fold (*P *< 0.05), 2.52-fold (*P *< 0.01) and 2.81-fold (*P *< 0.01), respectively, while STAT3 expression showed the increase of 2.32-fold (*P *< 0.001) and 2.07-fold (*P *< 0.05), respectively. Concurrently, the expression of the metabolism-related protein CYP7A1 was significantly increased by 3.03-fold (*P *< 0.05) compared with the Model group levels. The upregulation of these proteins (PCNA, Cyclin D1, Cyclin E1) indicates activated cell proliferation. Meanwhile, the increased phosphorylation of STAT3 and expression of YAP suggest the potential activation of the STAT3/YAP signaling pathway, which is closely associated with the liver regeneration process. Furthermore, the upregulation of the key BA metabolic enzyme CYP7A1 indicates that the SCHB nanoemulsion may support liver function recovery by regulating BA metabolic homeostasis.

### SCHB nanoemulsion promoted liver regeneration after PHx in the HCC mouse model

**Figure 8:**
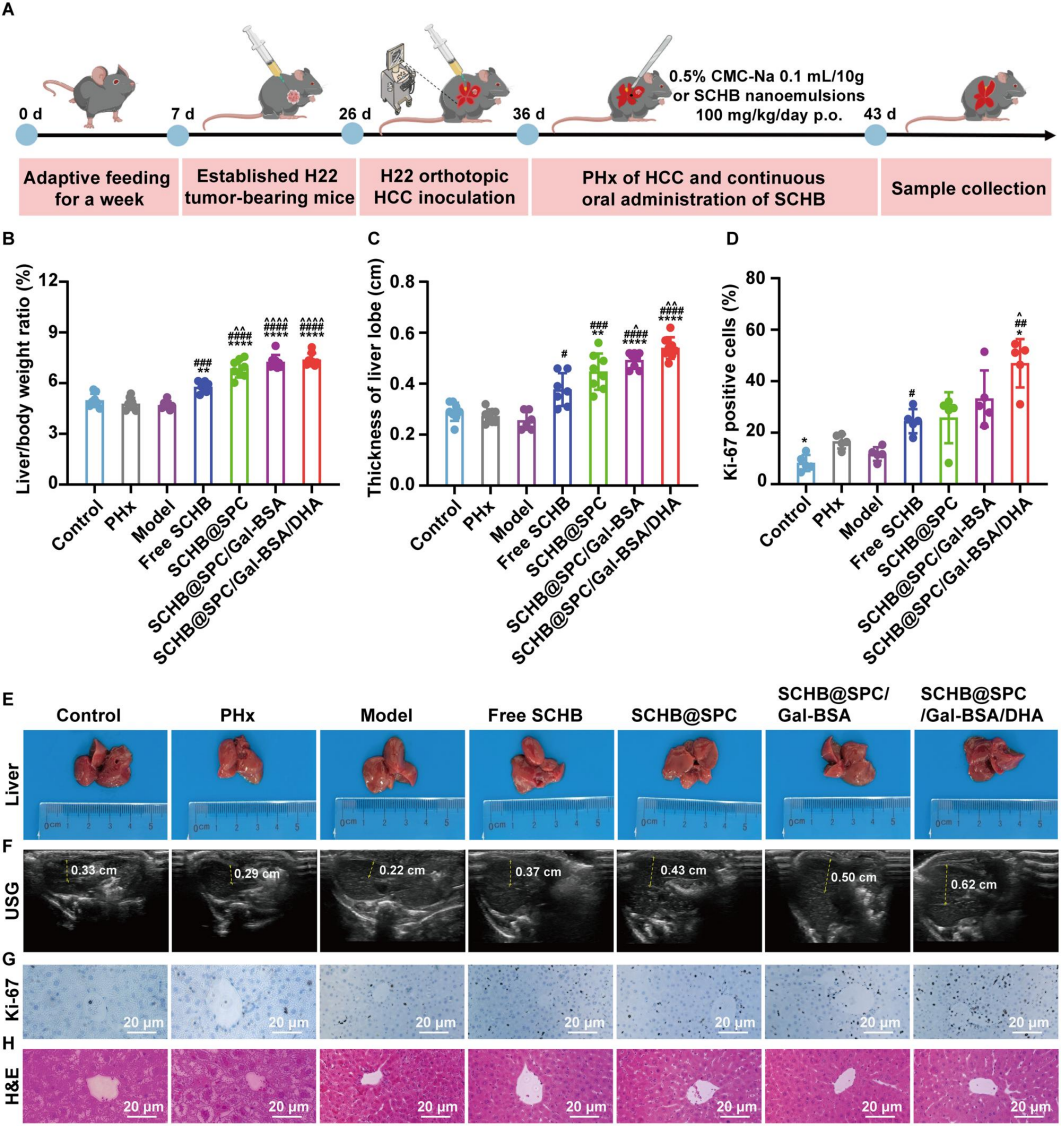
SCHB nanoemulsions promoted liver regeneration after *in situ* PHx in HCC mice. (**A**) Schematic of the animal treatment. (**B**) Liver/body weight ratios of mice (*n *= 8). (**C**) Statistical analysis of liver thickness data (*n *= 8). (**F**) USG images after PHx in HCC mice. The liver thickness was measured at the dotted line. Liver sections were stained with (**D** and **G**) Ki-67 and (**H**) H&E. Ki-67-positive cells in at least five microscope fields per sample (*n *= 5). Scale bar, 20 μm. (**E**) Liver on Day 7 after *in situ* PHx in HCC mice. **P < 0.01 and ****P < 0.0001 compared with the PHx; ^#^*P *< 0.05, ^###^*P *< 0.001 and ^####^*P *< 0.0001 compared with the model; and ^^^*P *< 0.05, ^^^^*P *< 0.01 and ^^^^^^*P *< 0.0001 compared with free SCHB. All data are presented as the mean ± SD.

A mouse xenograft model of *in situ* HCC was established, and tumor excision was performed to model liver regeneration under pathological conditions (Figure 8A and Supplementary Figure S20 and S21). Liver regeneration, as assessed by liver lobe thickness, was slower in the model group than in the PHx-only group. Mice treated with free SCHB or SCHB nanoemulsions after HCC resection showed increased liver lobe thickness, with the greatest increase observed in the SCHB@SPC/Gal-BSA/DHA group (Figure 8C and F). The liver/body weight ratio increased from 23.24% in the model group to a maximum of 57.36% in SCHB-treated groups. The SCHB@SPC/Gal-BSA/DHA group achieved the highest ratio of 7.38% (Figure 8B). The liver/body weight ratios in the SCHB nanoemulsion groups were significantly higher than in the free SCHB group. Representative liver images on Day 7 showed that livers in the SCHB@SPC/Gal-BSA/DHA group approached normal dimensions (Figure 8E).

Ki-67 staining was used to assess hepatocyte proliferation (Figure 8G). On Day 7, both the normal liver PHx group and the HCC *in situ* PHx groups showed notable cellular proliferation. Quantitative analysis indicated that free SCHB and SCHB nanoemulsion groups had higher percentages of Ki-67-positive cells than the PHx and model groups, with the SCHB@SPC/Gal-BSA/DHA group reaching 46.97% (Figure 8D). H&E staining revealed no liver damage or necrosis in SCHB nanoemulsion-treated groups, whereas the PHx group exhibited mild edema (Figure 8H).

**Figure 9:**
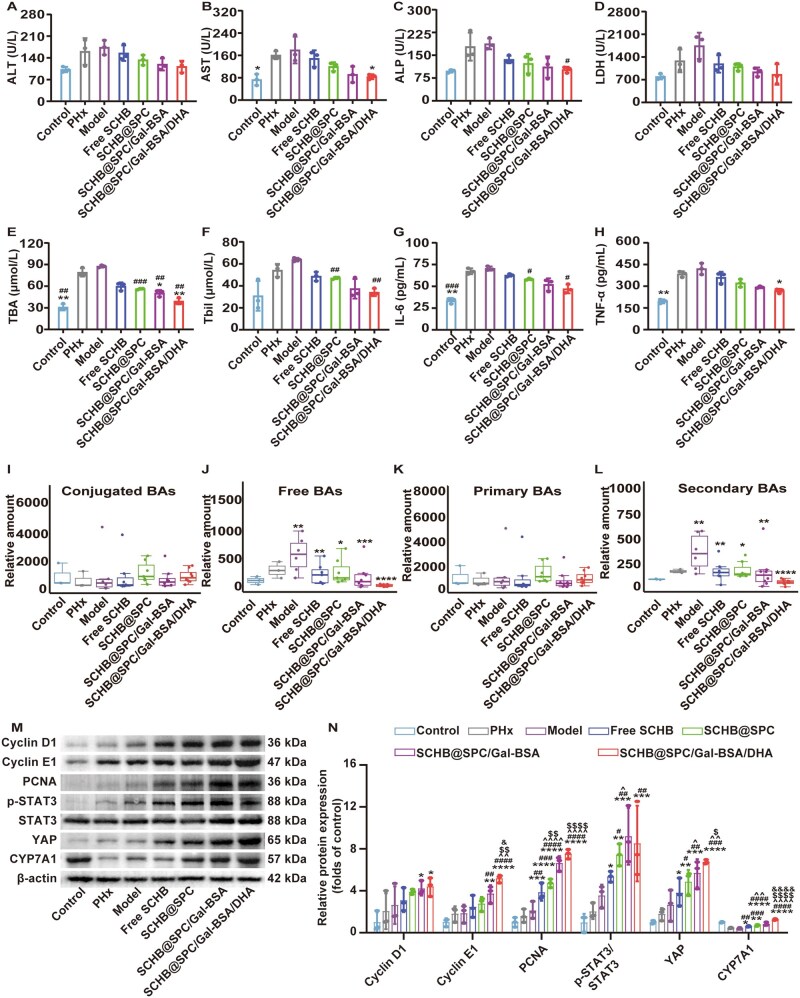
SCHB nanoemulsions promoted liver regeneration with PHx in HCC by promoting STAT3 phosphorylation and activating YAP. Serum levels of (**A**) ALT, (**B**) AST, (**C**) ALP, (**D**) LDH, (**E**) TBA, (**F**) Tbil, (**G**) IL-6 and (**H**) TNF-α in mice (*n *= 3). Relative amounts of (**I**) conjugated BAs, (**J**) free BAs, (**K**) primary BAs and (**L**) secondary BAs in the serum on Day 7 after PHx (*n *= 8). (**M** and **N**) Western blot analysis of Cyclin D1, Cyclin E1, PCNA, p-STAT3/STAT3, YAP and CYP7A1 on Day 7 after PHx (*n *= 3). *P < 0.05, **P < 0.01, ***P < 0.001 and ****P < 0.0001 compared with PHx; ^#^*P *< 0.05, ^##^*P *< 0.01, ^###^*P *< 0.001 and ^####^*P *< 0.0001 compared with the model; ^^^*P *< 0.05, ^^^^*P *< 0.01 and ^^^^^^*P *< 0.0001 compared with free SCHB; and ^$^*P *< 0.05, ^$$^*P *< 0.01 and ^$$$$^*P *< 0.0001 compared with SCHB@SPC; ^&^*P *< 0.05 and ^&&&&^*P *< 0.0001 compared with SCHB@SPC/Gal-BSA. All data are presented as the mean ± SD.

In the HCC mouse model after PHx, serum levels of ALT, AST, ALP and LDH were significantly elevated in the model group. Free SCHB and SCHB nanoemulsions reduced these elevated levels (Figure 9A–D). TBA and Tbil levels also increased after surgery, reaching 2.84- and 2.07-fold of control levels, respectively (Figure 9E and F). Treatment with free SCHB and SCHB nanoemulsions lowered TBA and Tbil levels, with the greatest reduction observed in the SCHB@SPC/Gal-BSA/DHA group. Levels of IL-6 and TNF-α were significantly increased in the PHx and model groups and decreased after SCHB treatment (Figure 9G and H). The SCHB@SPC/Gal-BSA/DHA group showed the most substantial decrease in these cytokines.

Serum concentrations of various BAs were assessed post-PHx in HCC mice. Treatment with free SCHB and SCHB nanoemulsions reduced serum levels of free BAs (e.g. alphaMCA and betaMCA) and secondary BAs (e.g. omegaMCA, DCA, UDCA and LCA). The most pronounced reduction was seen in the SCHB@SPC/Gal-BSA/DHA group. Conjugated and primary BAs showed no obvious changes (Figure 9I–L and Supplementary Figure S22).

Protein expression was measured by Western blotting after *in situ* PHx in HCC (Figure 9M and N). Consistent with PHx in healthy mice, after treatment with SCHB@SPC/Gal-BSA and SCHB@SPC/Gal-BSA/DHA, a significant increase in Cyclin E1 expression was observed by 2.01-fold (*P *< 0.01) and 2.80-fold (*P *< 0.0001), respectively. The three nanoemulsions increased the PCNA expression by 2.23-fold (*P *< 0.001), 3.13-fold (*P *< 0.0001) and 3.55-fold (*P *< 0.0001), respectively. Furthermore, following treatment with SCHB@SPC/Gal-BSA/DHA, YAP protein expression in the liver was markedly elevated by 1.83 (*P *< 0.05), 2.14 (*P *< 0.01) and 2.54 (*P *< 0.001) compared with that in the model group. In summary, enhanced liver regeneration induced by SCHB nanoemulsions following PHx treatment in HCC models was closely associated with increased hepatocyte proliferation. In addition, treatment with SCHB and different nanoemulsions resulted in a notable increase in CYP7A1 expression, particularly in the SCHB@SPC/Gal-BSA/DHA group, showing a 3.20-fold (*P *< 0.0001) increase. Consistent with the findings in the healthy 70% PHx model, the SCHB nanoemulsion, particularly SCHB@SPC/Gal-BSA/DHA, also upregulated the expression of cell cycle proteins (Cyclin E1, PCNA) and key molecules of the STAT3/YAP signaling pathway in this HCC model. This indicates its ability to activate similar pro-regenerative signaling axes even under pathological conditions. The significant upregulation of CYP7A1 further underscores its capacity to modulate BA metabolism in the diseased liver.

## Discussion

This study developed a dedicated nanoemulsion system to enhance the liver-targeted delivery of SCHB. The basic nanoemulsion (SCHB@SPC), prepared using SPC as a natural emulsifier, exhibited excellent drug loading capacity but suboptimal oral absorption, likely due to the intestinal epithelial barrier. To address this, we introduced Gal-BSA as a co-emulsifier to form SCHB@SPC/Gal-BSA, which improved emulsification performance and stability. Recognizing that TJs regulation is calcium-dependent [38], we further incorporated DHA, known to reversibly modulate TJs integrity [33, 39], resulting in the ternary SCHB@SPC/Gal-BSA/DHA system. This optimized nanoemulsion demonstrated excellent physicochemical properties, including remarkable stability and a hydrophilic surface (Figure 2K–M and Supplementary Figure S3), good *in vivo* biocompatibility (Supplementary Figures S5, S9, S15 and S16) and significantly enhanced oral bioavailability (Figure 5A and B), offering a new strategy for improving the intestinal permeability of lipid-based nanocarriers.

The SCHB@SPC/Gal-BSA/DHA nanoemulsion achieved a 7.64-fold higher liver accumulation and a 2.03-fold increase in absolute bioavailability compared to free SCHB (Figure 5A and C). This enhancement is attributed to efficient hepatocyte uptake, facilitated by galactose groups on Gal-BSA that specifically bind to ASGPR, mediating receptor-dependent endocytosis [40]. These properties enable the nanoemulsion system to not only overcome the intestinal absorption barrier but also achieve liver-specific targeting. *In vivo* experiments confirmed that compared with free SCHB, the nanoemulsion formulation significantly improved the oral bioavailability, highlighting its dual advantages in enhancing intestinal absorption and liver delivery.

In evaluating therapeutic efficacy, the 70% PHx model, a well-established system for studying liver regeneration [41], was employed. SCHB nanoemulsions significantly enhanced liver regeneration in both healthy mice subjected to 70% PHx and in mice with orthotopic HCC undergoing PHx. This was achieved through improved liver function and restoration of BAs metabolic homeostasis. While free SCHB treatment restored the liver/body weight ratio in PHx mice to near-control levels, the SCHB nanoemulsions, particularly SCHB@SPC/Gal-BSA/DHA, demonstrated superior efficacy in promoting regeneration without inducing pathological damage. This was evidenced by a higher rate of Ki-67-positive cells across all nanoemulsion groups (Figures 6C, E and 8D, G). Liver resection severely compromises hepatic function, reflected here by elevated serum levels of AST, ALT, ALP, Tbil and LDH, which are established markers of hepatocyte injury [42, 43]. Free SCHB moderately reduced these markers and supported liver repair, but SCHB nanoemulsions exhibited enhanced effects.

Mechanistically, the pro-regenerative effect of the SCHB nanoemulsion is closely related to its modulation of key signaling pathways. We observed that in both 70% PHx and orthotopic HCC mouse models, SCHB@SPC/Gal-BSA/DHA significantly enhanced the activation of the STAT3/YAP signaling axis and upregulated the expression of cell cycle regulatory proteins such as PCNA, Cyclin D1 and Cyclin E1 (Figures 7M, N and 9M, N). Concurrently, the nanoemulsion regulated BA metabolism by upregulating CYP7A1 expression, alleviating the adverse effects of postoperative BA metabolic disruption on liver regeneration, as shown by the reduction in serum levels of specific free and secondary BAs (Figures 7I–L and 9I–L). These results not only explain the mechanism by which SCHB promotes liver regeneration at the molecular level but also strengthen the causal link between functional recovery and the targeted delivery system, further supported by pharmacokinetic and pharmacodynamic correlations (Supplementary Figures S10–S13).

Functionally, the SCHB nanoemulsion demonstrated therapeutic effects superior to those of free SCHB across multiple aspects. In both healthy mice and HCC-bearing mouse models, the formulation significantly increased the liver/body weight ratio (Figures 6B and 8B), promoted the proportion of Ki-67-positive cells, improved liver function markers (e.g. ALT, AST, TBA, Tbil; Figures 7A–F and 9A–F) and reduced levels of pro-inflammatory cytokines TNF-α and IL-6 (Figures 7G, H and 9G, H). These consistent findings indicate that SCHB@SPC/Gal-BSA/DHA not only accelerates structural liver tissue regeneration but also promotes the recovery of metabolic and inflammatory status, offering multi-faceted therapeutic advantages.

This study confirms the efficacy of SCHB@SPC/Gal-BSA/DHA nanoemulsion in promoting liver regeneration in both healthy 70% PHx and orthotopic HCC PHx models. It is noteworthy that while the nanoemulsion significantly activated the STAT3/YAP signaling axis and upregulated cell cycle proteins (e.g. PCNA, Cyclin D1/E1) in both models, the underlying pathological context and potential challenges differ. In the healthy 70% PHx model, liver regeneration occurs in a relatively controlled microenvironment and the primary role of the nanoemulsion is to efficiently deliver SCHB to initiate and accelerate the endogenous regenerative program. In contrast, in the orthotopic HCC PHx model, the liver microenvironment is more complex, involving post-resection inflammatory storms, potential immunosuppression and possibly compromised function of the remaining hepatocytes. In this study, the nanoemulsion was equally effective in the HCC model in reducing inflammatory cytokine (TNF-α, IL-6) levels and ameliorating BA metabolic disorders, suggesting its ability not only to drive proliferation but also to mitigate the inhibitory effects of the pathological microenvironment on regeneration. Future studies could further delineate the precise signaling network differences mediating the efficacy of the nanoemulsion in these two models.

Although this study has made positive progress in exploring mechanisms and validating efficacy, certain limitations remain. For instance, the long-term stability of the nanoemulsion in the complex human gastrointestinal environment, the feasibility of industrial-scale production and its efficacy in non-human primates remain to be systematically evaluated. Future research should focus on the clinical translation pathway of this formulation, including standardization of the preparation process, stability studies and safety evaluations compliant with clinical standards, to facilitate its transition from the laboratory to clinical application.

In summary, this study confirms the potential of SCHB@SPC/Gal-BSA/DHA as an efficient oral liver-targeted nanodelivery system and provides solid preclinical evidence for its use in promoting liver regeneration after surgery. The development of this system offers valuable insights into the design of oral liver-targeted formulations and supports the clinical translation of SCHB.

## Conclusions

In summary, we have successfully developed an innovative oral liver-targeting nanoemulsion system, SCHB@SPC/Gal-BSA/DHA, which effectively overcomes the delivery challenge of SCHB and enhances liver regeneration post-hepatectomy. This nanoemulsion integrates SPC-based encapsulation, Gal-BSA-mediated liver targeting and DHA-enhanced intestinal absorption, resulting in favorable physicochemical properties, including a hydrophilic surface, uniform morphology and significant stability in storage and gastrointestinal environments. Safety evaluations confirmed its excellent biocompatibility *in vitro* and *in vivo*. The nanoemulsion significantly improved the oral bioavailability of SCHB and achieved preferential accumulation in the liver, as evidenced by a 7.64-fold higher hepatic concentration and a 2.03-fold increase in absolute bioavailability compared to free SCHB. Functionally, SCHB@SPC/Gal-BSA/DHA robustly promoted liver regeneration in both healthy and HCC-bearing mice subjected to 70% PHx or *in situ* PHx, as demonstrated by enhanced liver/body weight ratios, elevated Ki-67 positivity, improved serum biomarkers and activation of the STAT3/YAP signaling pathway. Importantly, beyond merely enhancing pharmacokinetics and liver targeting, our system directly addresses the core clinical bottleneck outlined in the Introduction. It achieves synchronous regulation of hepatocyte proliferation through the STAT3/YAP/Cyclin axis while maintaining metabolic homeostasis *via* CYP7A1-mediated BA normalization. This dual-action capability overcomes the limitation of conventional therapies that struggle to balance growth with function, thereby highlighting the unique translational value of our nanoemulsion platform. These findings not only validate the utility of a dual-targeting strategy for oral nanoemulsions but also establish SCHB@SPC/Gal-BSA/DHA as a promising therapeutic candidate for enhancing postoperative liver recovery in HCC patients. This study provides a robust preclinical foundation for the clinical translation of SCHB and offers a scalable platform for the design of liver-targeted oral nanomedicines.

## Supplementary Material

rbag019_Supplementary_Data

## Data Availability

All data required to evaluate the conclusions in the paper are available in the article itself and/or the Supplementary data. Requests for materials related to this study should be directed to the corresponding author and obtained through an Email.
