# Supplementary material for: Nanoemulsion based on natural components enables oral liver-targeting delivery of Schizandrol B to enhance liver regeneration after hepatectomy in hepatocellular carcinoma
Source: Regen Biomater. 2026 Feb 13;13:rbag019. doi: 10.1093/rb/rbag019 (PMC12988586; doi:10.1093/rb/rbag019)
Supplement: rbag019_Supplementary_Data [file rbag019_supplementary_data.docx]

**Supplementary materials**

**Nanoemulsion based on natural components enables oral liver-targeting delivery of schizandrol B to enhance liver regeneration after hepatectomy in hepatocellular carcinoma**

Yi Chen^1,2,†^, Jinzhuan Xu^1,†^, Jing Huang^1,†^, Huizhou He^1,3^, Zhimei Cheng^1,3^, Zhengli Zhou^1^, Man Zhang^1^, Yuanxing Huang^1^, Cao Huang^1^, Jianqing Peng^1,2^, Shuai Zhang^1,3,*^, Runbin Sun^4,*^, Zipeng Gong^1,*^

^1^ State Key Laboratory of Discovery and Utilization of Functional Components in Traditional Chinese Medicine, School of Pharmaceutical Sciences, Guizhou Medical University, Guiyang 561113, China

^2^ Guizhou Provincial Key Laboratory of Innovation and Manufacturing for Pharmaceuticals, Guizhou Provincial Engineering Technology Research Center for Chemical Drug R&D, Guiyang 561113, China

^3^ Department of Interventional Radiology, the Affiliated Hospital of Guizhou Medical University, Guiyang 550004, China

^4^ Phase I Clinical Trials Unit, Nanjing Drum Tower Hospital, Affiliated Hospital of Medical School, Nanjing University, Nanjing 210008, China

^*^ Corresponding authors. Email: zhangshuai476900@gmc.edu.cn (S.Z.); runbinsun@gmail.com (R.S.); gzp4012607@126.com (Z.G.)

^†^ These authors contributed equally to this work.

Supplementary materials total word count: **7437** words

**Supplementary Methods**

**Materials**

D-N-Acetylgalactosamine (Gal) was purchased from Hao Hong Biological Medicine Technology Co., LTD. (Shanghai, China). Schizandrol B (SCHB), bovine serum albumin (BSA, Grade V), and o-Phthalaldehyde (OPA) were purchased from Aladdin Reagent Inc. (Shanghai, China). Coomassie brilliant blue G250 was obtained from Sigma-Aldrich Inc. (St. Louis, MO, USA). Soybean phospholipids (SPC) were purchased from A.V.T Pharmaceutical Co., Ltd. (Shanghai, China). Cis-4,7,10,13,16,19-docosahexaenoic acid (DHA), nile red (NR), 1,1-dioctadecyl-3,3,3,3-tetramethylindotricarbocyaine (DiR), 3-hydroxyquinoline (HIQ), chlorpromazine, colchicine, and genistein were supplied by Macklin Biochemical Technology Inc. (Shanghai, China). Medium chain triglycerides (MCT) were supplied by Shinsun Pharmaceutical Inc. (Liaoning, China). Gastric mucin was provided by Dalian Meilun Biotech Inc. (Liaoning, China). DiO and DAPI were purchased from Beyotime Biotech Inc. (Shanghai, China).

Tumor necrosis factor-α (TNF-α) and Interleukin 6 (IL-6) enzyme-linked immunosorbent assay (ELISA) kits were supplied by ZCIBIO Technology Co., Ltd. (Shanghai, China). Detection kits for alanine transaminasealanine (ALT), aspartate aminotransferase (AST), total bile acid (TBA), total bilirubin (Tbil), alkaline phosphatase (ALP/AKP), and lactic dehydrogenase (LDH) were supplied by Nanjing Jiancheng Bioengineering Institute Inc. (Nanjing, China). Hematoxylin and eosin (H&E) stains, BCA protein assay kit, RIPA buffer (high), and phenylmethylsulfonyl fluoride (PMSF) were purchased from Solaibao Technology Co., LTD. (Beijing, China). ECL solution and RPMI 1640 medium were supplied by Thermo Fisher Scientific Inc. (PA, USA). Cell counting kit-8 (CCK-8) was obtained from APExBio Technology LLC. (Shanghai, China). Fetal bovine serum (FBS) was purchased from Cellmax (Cat SA201.02, Gansu, China). Penicillin-streptomycin solution and 0.25% TRYPSIN Solution (1×) were obtained from Saiaomei Cell Technology Co., LTD. (Beijing, China). The minimum essential medium (MEM) was purchased from Punosai Life Technology Co., LTD. (Wuhan, China). Anti-YAP, anti-PCNA, anti-Cyclin D1, and anti-β-actin antibodies were purchased from Cell Signaling Technology (CST, Shanghai, China), anti-Cyclin E1, anti-p-STAT3 and anti-STAT3 antibodies were purchased from Proteintech Group (Wuhan, China), anti-CYP7A1 antibody was purchased from Affinity Biosciences (Changzhou, China). Anti-ZO-1 protein antibody and goat anti-rabbit IgG H&L (Alexa Fluor®488) were purchased from Abcam Trading Co., Ltd. (Shanghai, China).

**Cells and animals**

Caco-2 cells were obtained from Procell Life Science & Technology Co., Ltd. (Wuhan, China) and maintained in MEM with 20% (v/v) FBS (Cellmax, Cat SA201.02), 1% penicillin, and streptomycin (P/S) (100 IU mL^−1^). AML12 cells and AML12 mouse liver parenchymal cell special medium, consisting of DMEM/F12 with 10% (v/v) FBS and 1% P/S (100 IU mL^−1^), were purchased from Shanghai Zhong Qiao Xin Zhou Biotechnology. H22 murine hepatoma cells were obtained from the Jinyuan Biotechnology Co., Ltd. (Shanghai, China) and were cultured in RPMI 1640 medium supplemented with 10% FBS and 1% P/S. These cells were cultured at 37℃ in a humidified 5% CO_2_ atmosphere.

The animal experiment procedures and animal ethics approval for this study were granted by the Animal Welfare and Ethics Committee of Guizhou Medical University (registration numbers 2302074 and 2303448). The animal experimental operations involved in the experiment were all in compliance with the requirements of animal ethics. Healthy C57BL/6 mice (male, 6-8 weeks, 22 ± 2 g) were provided by the Experimental Animal Center of Guizhou Medical University [Certificate No. SCXK-(Gui) 2023-0002] and bred in a standard animal laboratory environment for 1 week. All mice were housed under a 12 h light-dark cycle with free access to standard food and water at a temperature of 25℃ ± 1℃ and a humidity of 60% - 70%.

**Synthesis of Gal-BSA**

Gal-BSA was prepared *via* a green and safe Maillard reaction. In brief, BSA and Gal were dissolved in water at mass ratios of 1:1, 1:2, 1:3. After stirring at 25℃ for 12 h until completely dissolved, the solution was adjusted to pH values of 6, 7, and 8, and then lyophilized. The lyophilized powder was reacted at 60℃ and 79% humidity for 12, 24, 36, and 48 h in order to obtain Gal-BSA. The molecular weight distribution of Gal-BSA was analyzed using sodium dodecyl sulfate-polyacrylamide gel electrophoresis (SDS-PAGE). The degree of glycosylation (DG) and browning intensity (BI) of samples are typically used to reflect the progress of the Maillard reaction. Thus, the DG of Gal-BSA was evaluated by determining the decrease in free amino groups of the conjugates using the OPA method.

The DG was evaluated by determining the decrease of free amino groups of the conjugates by the OPA assay. In brief, the OPA reagent was freshly prepared before testing. Next, 0.2 mL of the BSA and Gal reaction solution at a concentration of 1 mg/mL was incubated with the OPA reagent (4 mL) at 35℃ for 2 min, and a mixture of 0.2 mL deionized water and 4 mL OPA reagent was used as the blank. Then, the absorbance of the solution was measured at 340 nm by a UV/Vis spectrophotometer. The above experiment was repeated 3 times. Under the same conditions, the absorbance of different concentrations of BSA solution and OPA reagent mixture was determined to establish the standard curve. The DG was evaluated by the following equation **(1)**:

$DG(\%)=\frac{C_{0}-C_{1}}{C_{0}}\times100$ **(1)**

Where *C*_0_ was the concentration of BSA, and *C_1_* was the concentration of Gal-BSA glycoconjugates.

The BI of Gal-BSA was analyzed according to a relative method with some modifications. Before measurement, the reaction solutions of Gal-BSA were diluted with deionized water to the concentration of 3 mg/mL, and absorbance of each sample at 294 nm and 420 nm was obtained by a UV/Vis spectrophotometer for intermediate and final products, respectively.

Take out the freshly prepared 30 µL emulsion at 0 min and dilute it 100 folds with 3 mL 0.1% SDS (w/v). After eddy mixing for 5 s, the absorbance at 500 nm was measured by a UV/Vis spectrophotometer and recorded as A_0_. Then take out the freshly prepared 30 µL emulsion at 10 min and dilute it with 3 mL 0.1% SDS (w/v) (1:100, v/v). After eddy mixing for 5 s, the absorbance at 500 nm was measured by a UV/Vis spectrophotometer and recorded as A_10_. Finally, the EAI and ESI of the emulsion were calculated using the following equations **(2)** and **(3)**:

$EAI(\frac{m^{2}}{g})=\frac{1\times2.303\times A_{0}\times N}{C\times\varphi\times L\times{10}^{4}}$ **(2)**

$ESI(\min)=\frac{A_{0}}{A_{0}-A_{10}}\times10$ **(3)**

Where *N* is the dilution factor (100), *C* is the concentration of BSA; *φ* and *L* are the oil phase volume fraction (0.25) and light path (1 cm), respectively. *A*_0_ and *A*_10_ are the absorbance of the emulsion at 0 min and 10 min, respectively.

**Preparation and optimization of SCHB@SPC/Gal-BSA**

The mass ratios of SPC (40 mg) to Gal-BSA were 1:0, 1:0.25, 1:0.5, and 1:0.75, respectively. The Gal-BSA was weighed and dissolved in deionized water to obtain the aqueous phase. Next, SCHB and SPC were mixed with 1 mL MCT to form an oil phase. The volume ratio of the aqueous phase to the oil phase was 4:1. The mixed solution was vortexed for 5 min, followed by 3 min of ultrasonication at 65 W. Then, the primary emulsion was passed through a microjet high-pressure homogenizer at a pressure of 6000 psi for three cycles to obtain nanoemulsion solutions loaded with SCHB. In addition, the stability of SCHB@SPC/Gal-BSA was evaluated by monitoring the changes in particle size (PS), polydispersity index (PDI), and zeta potential (ZP) for 7 d at 4℃ using dynamic light scattering (DLS).

**Preparation and optimization of SCHB@SPC/Gal-BSA/DHA**

SPC was mixed with DHA at varying concentrations (0, 10, 15, 20, 40, and 80 mM). SCHB@SPC/Gal-BSA/DHA was prepared as previously described. In brief, Gal-BSA was dissolved in deionized water as the aqueous phase, and then SPC and DHA were mixed with MCT to create an oil phase, with a volume ratio of aqueous to oil phase of 4:1. The preparation process parameters were previously described.

SCHB@SPC/Gal-BSA/DHA was prepared under optimal conditions as previously described. In brief, SPC and DHA were dissolved in 1 mL of SCHB/MCT solution at a concentration of 20 mM. Next, 4 mL of Gal-BSA solution was added to the mixture. The resulting solution was vortexed for 5 min and then ultrasonicated for 3 min at 65 W. The PS, PDI, and ZP of the preparations were measured using DLS. The loading efficiency (LE) and loading capacity (LC) of SCHB were determined using high-performance liquid chromatography (HPLC) and an ultraviolet detector (LC-16, Shimadzu Instruments Co., Ltd, Kyoto, Japan). SCHB content was detected at 254 nm using acetonitrile/water (65:35, v/v) as the mobile phase. The LE (%) and LC (%) of the SCHB were calculated using the following equations **(4)** and **(5)**:

$LE(wt.\%)=\frac{weight of loaded drug}{weight of total drug}\times100$ **(4)**

 $LC(wt.\%)=\frac{weight of loaded drug}{theoretical total weight of nanoemulsion}\times100$ **(5)**

The appearance of SCHB@SPC/Gal-BSA/DHA was examined using transmission electron microscopy (TEM) (H-7800, Hitachi Co., Ltd., Tokyo, Japan). The saturation solubility of SCHB in simulated gastric fluid (SGF) and simulated intestinal fluid (SIF) were measured, and the release of SCHB from SCHB@SPC, SCHB@SPC/Gal-BSA, and SCHB@SPC/Gal-BSA/DHA into SGF and SIF were evaluated.

The optimal ratio of SPC to DHA was evaluated by estimating the ability of SCHB@SPC/Gal-BSA/DHA to open TJs of the Caco-2 cell monolayer. Firstly, the uptake of Caco-2 cells was detected. In conclusion, Caco-2 cells were inoculated into 6-well plates at a density of 1 × 10^4^ cells/well, and the corresponding nanoemulsions were given the different DHA contents (0, 10, 15, 20, 40, 80 mM), respectively, and incubated with the nanoemulsions for 4 h. Next, the plates were washed with PBS, digested with pancreatic enzyme, and collected cells. The cells were lysed with RIPA high-efficiency cell lysate for 1 h, and the protein content of Caco-2 cells was quantified by a BCA protein quantification kit. The cellular uptake of SCHB was determined by HPLC, and the P_app_ of SCHB@SPC/Gal-BSA/DHA at different ratios was detected. Briefly, the Caco-2 monolayers were washed twice with PBS. Then 0.2 mL free SCHB, SCHB@SPC, SCHB@SPC/Gal-BSA and SCHB@SPC/Gal-BSA/DHA, SCHB@SPC/Gal-BSA/DHA (the content of DHA at 0, 10, 15, 20, 40, 80 mM) (100 μg/mL SCHB) were added to the upper chambers, adding 0.6 mL PBS to the lower chambers. The transepithelial electrical resistance (TEER) of Caco-2 cell was determined to investigate the effect of the nanoemulsions on the TJs of Caco-2 cell monolayer membrane.

**Immunofluorescence**

Caco-2 cells were cultured on a sterilized tablet in a 12-well plate and fused to about 70%. The Caco-2 cells were subsequently cultured in a medium devoid of antibiotics and serum for 24 h, followed by treatment. The SCHB nanoemulsions were added and incubated at 37℃ (5% CO_2_) for 2 h. At the end of the incubation period, the Caco-2 cells on coverslips were rinsed with preheated PBS for 5 min, 3 times. Next, the cells were fixed with 4% paraformaldehyde at room temperature for 20 min, and then rinsed with PBS for 5 min, three times; subsequently, the cell monolayer was treated with 0.1% Triton X-100 in 0.01 M PBS for 10 min to enhance membrane permeability. In addition, the cells were blocked with PBST (PBS + 0.1% Tween 20) containing 1% BSA and 22.52 mg/mL glycine at room temperature for 30 min, followed by rinsing with PBS for 5 min, 3 times. And, recombinant Anti-ZO-1 protein antibody (antibody dilution 1:100), dropped on a slide to cover cells, incubate at 4℃ overnight. Put the sample at room temperature for 15 min, and use preheated PBS to rinse for 5 min for 3 times; Goat Anti-Rabbit IgG H&L (Alexa Fluor®488) (Antibody dilution 1:1000) incubated at room temperature for 1 h away from light; 1 μg/mL DAPI incubated at room temperature for 10 min away from light; Rinsed with PBS for 5 min for 3 times, then put a drop of anti-fluorescence quenching on the cleaning slide invert it on the slide, and apply two drops of nail polish to the edge and dry at room temperature away from light, protected at -20℃. Finally, laser confocal microscopy (LSM 900, Zeiss) showed that the color was green after excitation by 488 nm.

***In vitro* drug release of SCHB-loaded nanoemulsions**

SGF and SIF were prepared as follows. To make SGF, 16.4 mL diluted hydrochloric acid (equivalent to 3.84 mL hydrochloric acid) and 10 g pepsin were dissolved in 800 mL water and then water was added to dilute to 1000 mL. To make SIF, 6.8 g potassium dihydrogen phosphate was dissolved in 500 mL water, and the pH value was adjusted to 6.8 with 0.1 mol/L sodium hydroxide solution. Subsequently, 10 g of pancreatic enzyme was added to some water to dissolve, and then the two liquids were mixed and diluted to 1000 mL with water. To study the dynamic release of SCHB@SPC, SCHB@SPC/Gal-BSA, and SCHB@SPC/Gal-BSA/DHA in GIT, the study was performed with membrane dialysis technique in SGF for 2 h and then nanoemulsions were transferred to SIF for 24 h. In brief, 1 mL of the nanoemulsions were placed in a dialysis bag (molecular weight cut-off, 3.5 kDa) and dispersed in 20 mL of release medium with 100 rpm/min for 37℃ at the setting time. The 0.5 mL of release medium was absorbed at a predetermined time point, and 0.5 mL of fresh release medium was added. The release of SCHB in the medium was analyzed by HPLC. All the drug release studies met the requirements of precipitation conditions.

**The determination of the saturation solubility of SCHB in artificial gastroenteric fluid**

SGF and SIF were prepared according to the prescription described above. Excessive SCHB was added into 10 mL SGF and SIF solutions, and dissolved by stirring while adding SCHB until SCHB was no longer dissolved. The solution was oscillated in a constant temperature oscillator at 37℃ for 24 h. After the supersaturated solution was oscillated for 24 h, the solution was centrifuged (3000 rpm, 10 min). The supernatant was analyzed by HPLC and the saturation solubility of SCHB in the artificial gastroenteric fluid was calculated.

**Stability of @SPC/Gal-BSA/DHA in various simulated media**

In short, SPC, DHA, NR, and HIQ were dissolved in MCT to form an oil phase. Gal-BSA was dissolved in deionized water as the water phase. The water and oil phases were mixed in a volume ratio of 4:1. The mixed solution was vortexed for 5 min and followed with sonication in an ice-water bath at 65 W for 3 min to obtain primary emulsion. Then, the primary emulsion was passed through a micro-injection high-pressure homogenizer at 6000 psi for 3 cycles to obtain a nanoemulsion solution loaded with fluorescein.

Degradation of @SPC/Gal-BSA/DHA in the gastrointestinal tract (GIT) was also investigated. HIQ/NR@SPC, HIQ/NR@SPC/Gal-BSA, and HIQ/NR@SPC/Gal-BSA/DHA were prepared using the Förster resonance energy transfer (FRET) pair of HIQ and NR. The nanoemulsions were then mixed with SGF or SIF in tubes. Next, the mixtures were placed in a shaking incubator (100 rpm/min, 37℃ ± 0.5℃) at 37℃ for 0, 0.5, 1, 2, 4, 6, 8, and 12 h. The fluorescence intensities of the samples were measured using a fluorescence spectrophotometer (Cary Eclipse, VARIAN, US). The FRET ratio (FR) was calculated using the following equation **(6)**:

$FR(\%)=\frac{I_{NR}}{I_{NR}+I_{HIQ}}\times100$ **(6)**

where *I_HIQ_* and *I_NR_* are the fluorescence intensities of HIQ and NR at 510 and 635 nm, respectively, and the excitation wavelength is 450 nm.

**Transcellular permeation evaluation in the Caco-2 cell monolayer**

To investigate the transcellular permeability of SCHB in monolayers, Caco-2 cells were used as an *in vitro* model of the gastrointestinal epithelium. In addition, Caco-2 cell viability in the SCHB nanoemulsions was assessed. Caco-2 cells were seeded at a cell density of 5 × 10^4^ cells per well on permeable transwell inserts (membrane area, 0.33 cm^2^; pore size, 0.4 μm), and these inserts were placed on 24-well culture plates for 21 d. The cells were cultured in MEM containing 20% (v/v) FBS until they became confluent and polarized. The TEER is a method used to measure ion flow through a single layer of cells to assess the viability of epithelial barrier function. The TEER across the Caco-2 cell monolayer was measured using a voltammeter (Millicell ERS-2, Millipore Co., Ltd., MA, USA) at the start and end of the experiments. When the TEER values reached 500 Ω·cm^2^, the cell monolayers were used in the following experiment. Next, to prepare samples, free SCHB, SCHB@SPC, SCHB@SPC/Gal-BSA, and SCHB@SPC/Gal-BSA (100 μg/mL SCHB) were diluted with MEM without FBS. Free SCHB was formulated using dimethyl sulfoxide (DMSO), and the concentration of DMSO in the final solution did not exceed 0.1%. The Caco-2 monolayers were washed twice with PBS. Then, 0.2 mL SCHB nanoemulsions were added to the upper chambers, and 0.6 mL PBS was added to the lower chambers. After incubation for 0, 0.5, 1, and 2 h, the TEER values of Caco-2 cells were determined to investigate the effect of the nanoemulsions on the TJs of the Caco-2 cell monolayer membranes. After 2 h of incubation, 0.5 mL samples were collected from the lower chambers, and methanol was added to extract SCHB. The SCHB content was determined using HPLC, and the apparent permeability coefficient (P_app_) was calculated. After 2 h, parts of the apical and basolateral media were collected and observed using TEM. The P_app_ of SCHB was calculated using the following equation **(7)**:

$P_{app}=\frac{Q}{AC_{0}t}$ **(7)**

where *Q* is the cumulative amount (μg) of the SCHB transported into the lower chambers, *A* is the Caco-2 cell monolayer area (cm^2^), *C*_0_ is the initial SCHB concentration (μg/mL) in the upper chambers, and *t* is the duration (s) of the permeability experiment.

**Preparation of DiO and DiR nanoemulsions**

In brief, SPC, DHA, and DiO or DiR were dissolved in MCT to form an oil phase. Gal-BSA was dissolved in deionized water as the water phase. The water and oil phases were mixed in a volume ratio of 4:1. The mixed solution was vortexed for 5 min and followed with sonication in an ice-water bath at 65 W for 3 min to obtain primary emulsion. Then, the primary emulsion was passed through a micro-injection high-pressure homogenizer at 6000 psi for 3 cycles to obtain a nanoemulsion solution loaded with fluorescein.

**Assessment of intestinal distribution and liver-targeting capability of DiR-labeled SCHB nanoemulsions**

C57BL/6 mice were fasted overnight and then randomized into four groups (*n* = 3). DiR/SCHB nanoemulsions (free DiR, DiR/SCHB@SPC, DiR/SCHB@SPC/Gal-BSA, or DiR/SCHB@SPC/Gal-BSA/DHA) were prepared, and the mice were administered orally by gavage with DiR nanoemulsions containing 2 mg/kg DiR and 100 mg/kg SCHB. After treatment for 0.5, 1, 4, 8, and 12 h, the animals were imaged using an *in vivo* imaging spectrum system (IVIS) (Lumina III, Caliper, Signal Hill, CA, USA) equipped with small animal anesthetic equipment. Next, the mice were sacrificed, and the major tissues, including the heart, liver, spleen, lung, and kidney, were isolated for imaging. Furthermore, the GITs of the mice were isolated to observe the distribution of the nanoemulsions. After appropriate processing, the fluorescence intensities of the tissues and intestines were analyzed with Living Image®4.5.5 software.

**Distribution of @SPC/Gal-BSA/DHA in the GIT**

DiO/SCHB nanoemulsions were prepared to investigate the distribution of @SPC/Gal-BSA/DHA in the GIT. Male C57BL/6 mice were randomly divided into four groups (*n* = 3) and administered orally by gavage with free DiO, DiO/SCHB@SPC, DiO/SCHB@SPC/Gal-BSA, or DiO/SCHB@SPC/Gal-BSA/DHA (3 mg/kg DiO and 100 mg/kg SCHB). The mice were sacrificed at 0.5, 1, 4, 8, and 12 h after administration. The ileum was collected, opened, and gently rinsed with PBS. After fixation in 4% paraformaldehyde, paraffin sections were prepared and stained with DAPI. Finally, imaging was performed using CLSM (LSM 900, Zeiss).

**Pharmacokinetic study of SCHB nanoemulsions**

For pharmacokinetic analysis, seventy-two healthy male C57BL/6 mice were randomly divided into four groups (*n* = 18). The free SCHB (p.o.), SCHB@SPC, SCHB@SPC/Gal-BSA, or SCHB@SPC/Gal-BSA/DHA were administered orally by gavage at an SCHB dose of 100 mg/kg. Furthermore, at the same time, free SCHB (i.v.) was administered *via* tail vein injection. Every group was further randomly assigned to three sub-groups (*n* = 6). The mice in each group were anesthetized using inhaled isoflurane with a small animal anesthetic equipment (ZS-MV-IV, Zhongshi Scientific Instruments Co., Ltd., Beijing, China). Blood samples were collected from the lateral tail veins of mice under anesthesia at 0, 0.25, 2, 8 h for sub-group one, 0.083, 0.5, 4, 12 h, for sub-group two, and 0.17, 1, 6, 24 h, for sub-group three after oral administration. The samples were centrifuged at 1000 ×g and 4℃ for 10 min. Plasma was collected and stored at -80℃ for further analysis. To prepare plasma samples, 30 μL of mouse plasma was combined with 300 μL of methanol containing the internal standard (IS, berberine, 100 ng/mL) working solution. The samples were shaken for 3 min and centrifuged at 11 000 ×g for 5 min. Then, 200 μL of the supernatant was transferred to a new tube and centrifuged again at 11 000 ×g for 5 min, and 5 μL of the resultant supernatant was injected for LC-MS/MS analysis. LC-MS/MS analysis (SCIEX 4000 mass spectrometer, Sciex, CA, USA) coupled with Shimadzu LC-20 HPLC system (Shimadzu, Japan). The MS parameters were as follows: ion spray voltage, 5500 V (positive mode); source temperature, 550℃; curtain gas, 30 psi; nebulizer gas, 50 psi; and heater gas, 60 psi. The MRM transitions of SCHB and IS were 399.2→368.2 and 336.4→320.2, respectively. The chromatographic separation was performed using the Zorbax C18 column (50 × 2.1 mm, 3.5 μm, Agilent, Santa Clara, CA, USA). The mobile phase consisted of water containing 0.1% formic acid, 5 mM ammonium acetate (mobile phase A), and acetonitrile (mobile phase B). The gradient elution was programmed as follows: the mobile phase B was maintained at 20% from 0.00 to 1.00 min, increased from 20% to 90% between 1.00 and 3.00 min, maintained at 90% from 3.00 to 5.00 min, decreased from 90% to 20% from 5.00 to 5.10 min, and finally maintained at 20% from 5.10 to 7.00 min at a flow rate of 0.4 mL/min. The temperature of the column and autosampler was controlled at 40℃ and 4℃, respectively. The pharmacokinetic parameters of SCHB were analyzed using WinNonlin 7.0 (Pharsight, Mountain View, CA, USA).

***In vivo* biodistribution of SCHB nanoemulsions**

Ninety-six Male C57BL/6 mice were used to investigate the *in vivo* biodistribution of SCHB nanoemulsions. Mice were randomized into four groups (*n* = 24). Free SCHB, SCHB@SPC, SCHB@SPC/Gal-BSA, and SCHB@SPC/Gal-BSA/DHA were administered orally by gavage at an SCHB dose of 100 mg/kg. To evaluate the biodistribution of SCHB, heart, liver, spleen, lungs, and kidney samples were obtained from mice under anesthesia at 0.5, 1, 4, and 8 h after dosing using small animal anesthetic equipment. The tissue samples were washed with normal saline to remove blood, dried using filter paper, and weighed (*n* = 6). For analysis, approximately 50 mg of tissue was weighed and homogenized with 1 mL of 80% methanol containing the IS (100 ng/mL) working solution. The samples were centrifuged at 11 000 ×g for 5 min. Then, 200 μL of the supernatant was transferred to a new tube and centrifuged at 11 000 ×g for 5 min. Finally, 5 μL of the resultant supernatant was injected for LC-MS/MS analysis.

The uptake of SCHB by AML12 cells was measured using HPLC. Firstly, the uptake of SCHB in AML12 cells was detected. AML12 cells were inoculated into 6-well plates at a density of 8 × 10^3^ cells/well, and the SCHB nanoemulsions were added, and incubated with the nanoemulsions for 4 h. Next, the plates were washed with PBS, digested with pancreatic enzyme, and the cells were collected. The cells were lysed with RIPA (high-efficiency) cell lysate for 1 h, and the protein content of AML12 was quantified using a BCA protein quantification kit. The cellular uptake of SCHB was determined by HPLC.

**Pharmacokinetic modeling correlating nanoemulsion parameters with hepatic uptake and regeneration efficiency**

To elucidate the impact of formulation design on liver regeneration efficacy (e.g., liver-to-body weight ratio), we analyzed the correlations between key physicochemical parameters of the nanoemulsion (e.g., PDI, PS, and ZP) and its hepatic uptake, along with serum and hepatic AUC using PK/PD modeling (Multiple Linear Regression Model) by R project (version 4.4.1).

**The cell viability of SCHB nanoemulsions on Caco-2 and AML12 cells**

To evaluate cell viability, Caco-2 and AML12 cells were selected as cell models. The cell viability study of free SCHB and SCHB@SPC/Gal-BSA/DHA was analyzed by CCK-8 assay *in vitro*. In a word, Caco-2 and AML12 cells were seeded in 96-well culture plates at a density of 8 × 10^3^ cells/well. After the cells reached 70% - 80% fusion, they were treated with different SCHB concentrations of 2, 4, 8, 16, 32, 64, 128, and 256 μg/mL (0.1% DMSO) and other nanoemulsions blank carrier with SPC concentration of 10 - 80 μg/mL for 24 h. The control group was treated with an empty medium solution (without any samples). After incubation, 100 µL of CCK-8 solution was added to each well, followed by incubation at 37℃ for 4 h. Finally, the cell viability was analyzed with a microplate reader (EPOCH2, Agilent BioTek, USA) at a wavelength of 450 nm. The cell viability was calculated as follows equation **(8)**:

$Cell viability (\%)=\frac{{OD}_{detected}-{OD}_{blank}}{{OD}_{positive}-{OD}_{blank}}\times100$ **(8)**

**70% PHx** **model**

The 70% PHx model in mice represents the most commonly used model for the study of liver regeneration to understand the underlying molecular mechanisms. PHx was performed according to the standard procedure as previously described [1]. In brief, the mice were induced anesthesia with 3% - 4% isoflurane for about 30 s and maintained anesthesia with 1% - 2% isoflurane until the end of the experiment. Next, the mice were shaved in the abdominal area using an appropriate electric fur shaver, and then fixed to the operating board in an X-shaped supine position using tape and disinfected the skin with 75% ethanol. Make a midline abdominal skin and muscle incision (about 3 cm long) to expose the xiphoid process. Then, the left anterior lobe was ligated with absorbable sutures (4-0, Pudong Jinhuan Medical Supplies Co., LTD, Shanghai, China), and the parenchymal tissue distal to the ligature was resected. Subsequently, the right anterior lobe and the left posterior lobe were ligated with sutures and resected between the gall bladder and the suprahepatic vena cava. Then, we needed to check carefully for the presence of bleeding points using a cotton swab: minor bleeding could occur from the resected lobe and wash the intraperitoneal cavity and organs with warm saline. After surgery, the peritoneum and outer skin were sewn together, the incision was closed by using an absorbable suture, and the skin surrounding the suture was wiped with 75% ethanol. Lastly, mice were kept in a 37℃ environment until awake before transferring into the cages. The PHx procedures were completed within 10 - 15 min. Postoperative management: on one hand, temperature control: to prevent hypothermia of mice under anesthesia, the mice were placed on heating pads during the entire operation. On the other hand, incision management: After closing the peritoneum, the incision was cleaned to reduce abdominal irritation.

In the process of maintaining consistency of the 70% PHx model, we mainly control the following aspects. The first was the surgical technique: In mice, 70% PHx was achieved by removing the left lateral lobe and middle lobe, which made up about 70% of the total liver. Therefore, we first calculated the ratio of the weight of the removed liver lobe to the body weight at the time of sacrifice through pre-experiment to ensure the establishment of the 70% PHx model. The experimental operation was stable and reliable. During the operation, the method of ligation of the anatomic functional units of the liver was used step by step to ensure the accuracy and consistency of the operation.

**Evaluation of SCHB nanoemulsions on liver regeneration in the 70% PHx model**

Forty-two healthy male C57BL/6 mice were randomly divided into the control, model, free SCHB, SCHB@SPC, SCHB@SPC/Gal-BSA, and SCHB@SPC/Gal-BSA/DHA groups (*n* = 7 per group). The safety of SCHB nanoemulsions was evaluated before the efficacy test. All mouse groups, except the control group, received 70% PHx. SCHB was suspended in 0.5% (w/v) sodium carboxymethyl cellulose (CMC-Na) solution as previously described. To implement blinding, the personnel who prepared the formulations were different from those who performed the animal dosing. The mice in the control and model groups were treated with a 0.5% CMC-Na solution, whereas those in the other four groups were treated with 100 mg/kg SCHB nanoemulsions. All mice were administered orally by gavage 5 min after surgery and once daily until the end of the study. The mice were then sacrificed at the indicated time points (0, 1, 3, 5, and 7 d). After treatment, blood samples and liver tissues were harvested. Blood samples were centrifuged at 1000 ×g for 10 min, and the serum was collected. Liver/body weight ratios were calculated using the liver and body weights of the animal at the time of sacrifice. The middle part of the right posterior liver lobe was immediately fixed in 4% paraformaldehyde for H&E staining and immunohistochemistry, and other liver tissues were prepared for Western blot analysis. Serum was then used to evaluate liver function, BA metabolic levels, and inflammatory factor levels. The serum and remaining tissues were stored at -80℃ for further use.

**Construction of the C57BL/6 mice H22 HCC orthotopic transplantation model**

Primarily, the frozen H22 murine hepatoma cells were thawed and passed through more than 3 generations. Then the cells were inoculated into the abdominal cavity of mice for the construction of the model of tumor ascites. After 7 d of inoculation, the abdominal fluid of mice was extracted and the cell density was adjusted to 1 × 10^7^/mL. H22 cells from ascites were collected and then inoculated into the skin of male C57BL/6 mice for tumor-bearing. After 12 d, the tumor tissue was collected and cut into tissue blocks for standby application. Then, H22 HCC transplanted by *in situ* transplantation in the C57BL/6 mice was constructed. In brief, mice were anesthetized with isoflurane. The mice were placed in a supine position and fixed on the experimental plate. After that, a disposable sterile medical pad was placed, and the skin was disinfected with iodine. The skin and peritoneum were cut layer by layer along the midabdominal line under the xiphoid process to fully expose the left lobe of the liver. 50 μL of tissue blocks were extracted with a 50 μL microsyringe, and the tumor tissue blocks were slowly injected into the liver parenchyma at a 20˚ angle of about 0.5 cm. After the injection, the needle was removed, and the needle hole was immediately pressed gently with sterile gauze to the liver surface to stop bleeding, and the abdomen was closed layer by layer. After the operation, keep the rewarming in time, and then continue to feed, eat freely, and water. Subsequently, after about 10 d, liver tumor formation was determined by ultrasonography (USG), indicating the successful generation of the orthotopically implanted liver tumor model, conforming to the successful establishment of the H22 HCC transplanted by the *in situ* transplantation model.

**PHx of H22 HCC orthotopic transplantation model**

The PHx of the H22 HCC orthotopic transplantation model was performed with the left lobe of the liver resected according to the PHx procedure mentioned above. Briefly, the mice were induced and maintained under anesthesia with isoflurane until the end of the experiment. Next, the mice were fixed to the operating board and the skin was disinfected with 75% ethanol. Make a midline abdominal skin and muscle incision (about 3 cm long) to expose the xiphoid process. The left lobe of the liver inoculated with the H22 HCC was then ligated and resected. After surgery, taking the peritoneum and outer skin together, and using an absorbable 4-0 silk to close the incision, mice were kept in a heating environment until awake before transferring into the cages.

**Evaluation of SCHB nanoemulsions on liver regeneration in in situ PHx of HCC**

H22 cells were transplanted *in situ* into the liver of normal mice for 10 d, and USG was performed to confirm the establishment of the orthotopic HCC mouse model using a high-grade color Doppler ultrasound instrument (LOGIQ E11 CE, New York, NY, USA) with a linear array probe (frequency L8-18i). PHx was then performed in mice with *in situ* HCC. Moreover, healthy control and healthy PHx groups were established (*n* = 8 per group). Forty HCC PHx mice were randomly divided into five groups: model, free SCHB, SCHB@SPC, SCHB@SPC/Gal-BSA, and SCHB@SPC/Gal-BSA/DHA (*n* = 8 per group). The mice then received oral administration of 0.5% CMC-Na, free SCHB, and SCHB nanoemulsions at an SCHB dose of 100 mg/kg once daily for 7 d. To implement blinding, the personnel who prepared the formulations were different from those who performed the animal dosing. USG was used to monitor tumor growth, and tumors approximately 0.5 cm in diameter were selected for surgical resection. At the end of treatment, blood and liver tissue samples were collected for follow-up tests, including liver/body weight ratio assessment, histological analysis, liver function tests, inflammatory and metabolic level evaluation, and Western blot analysis.

**Serum biochemical evaluation**

The activity levels of serum ALT, AST, ALP, TBA, Tbil, and LDH were analyzed by using biochemical test kits from Nanjing Jiancheng Bioengineering Research Institute Co., LTD.

**Evaluation of inflammatory cytokines in serum**

IL-6 and TNF-α are vital inflammatory factors in liver regeneration following PHx. The cytokines expression of cytokines TNF-α and IL-6 following PHx in the serum after treatment were measured using ELISA kits as directed by the manufacturer. The absorbance was detected by a microplate reader at 560 nm.

**Histological analysis**

For histology evaluation, tissues were immediately fixed in 4% paraformaldehyde, embedded in paraffin wax, sectioned, and stained for H&E and Ki-67. The Ki-67 expression was detected by immunostaining with previous Ki-67 antibodies according to a standard protocol-reported method to monitor hepatocyte proliferation. Ki-67-positive hepatocytes were counted in at least 5 microscopic fields (40× magnification) for each sample to determine the number of proliferating hepatocytes. The H&E and Ki-67 staining were observed by a Fluorescent inverted microscope (TI-U, Nikon Corporation, Japan) and the proportion of Ki-67 positive cells was detected by Qupath®0.5.1.

**Measurement of BAs**

To evaluate the effects of SCHB and SCHB nanoemulsions on the BA profile in mouse serum, different BAs were measured using LC-MS/MS (SCIEX 4000 mass spectrometer, Sciex, CA, USA). BAs were analyzed using a previously described method [2]. In brief, 50 μL of plasma was mixed with 200 μL of methanol containing the IS working solution (Cholic-2,2,4,4-D_4_ acid, d_4_-CA, 100 ng/mL). Samples were shaken for 3 min and centrifuged at 11 000 ×g for 5 min. Finally, 200 μL of the supernatant was transferred to a new tube and centrifuged again at 11 000 ×g for 5 min, after which 5 μL of the final supernatant was injected for LC-MS/MS analysis. The MS parameters were as follows: ion spray voltage, -4500 V (negative mode); source temperature, 550℃; curtain gas, 30 psi; nebulizer gas, 50 psi; and heater gas, 60 psi. Chromatographic separation was performed using a Waters Atlantis T3 column (100 × 2.1 mm, 3 μm). The mobile phase comprised water containing 0.1% formic acid (mobile phase A) and methanol (mobile phase B). The gradient elution was programmed as follows: the mobile phase B was maintained at 60% from 0.00 to 3.00 min, increased from 60% to 90% between 3.00 and 10.00 min, maintained at 90% from 10.00 to 15.00 min, decreased from 90% to 60% from 15.00 to 15.10 min, and finally maintained at 60% from 15.10 to 20.00 min at a flow rate of 0.2 mL/min. The temperature of the column and autosampler was controlled at 40℃ and 4℃, respectively.

**Western blot analysis of proteins in the liver**

The total protein of the liver in each group was extracted with RIPA lysis buffer containing 0.1 mmol/L phenylmethanesulfonyl fluoride (PMSF). Protein levels were measured using a BCA assay kit (Beyotime Biotechnology, Shanghai, China). The protein extracts were then mixed with loading buffer at a ratio of 4:1 and heated at 95℃ for 10 min. The protein samples were subjected to SDS-PAGE and transferred to a 0.45-μm polyvinylidene difluoride membrane (PVDF). After adding 5% skim milk, the membranes were immunoblotted with anti-Cyclin D1 (1:1000), anti-Cyclin E1 (1:1000), anti-PCNA (1:1000), anti-p-STAT3 (S727) (1:1000), anti-STAT3 (1:1000), anti-YAP (1:1000), anti-CYP7A1 (1:1000), and anti-β-actin (1:2000) with gentle shaking at 4℃ overnight. After three rinses with PBST (10 min each), the membranes were incubated with horseradish peroxidase (HRP)-conjugated goat anti-rabbit IgG secondary antibodies (1:5000). The intensity of the bands was quantified using Image Laboratory statistical software (Bio-Rad Laboratories, Hercules, CA, USA).

**Safety evaluation**

C57BL/6 mice were randomly divided into the control group (0.5% CMC-Na), 70% PHx model group (0.5% CMC-Na), free SCHB group (100 mg/kg), SCHB@SPC group (100 mg/kg), SCHB@SPC/Gal-BSA group (100 mg/kg), SCHB@SPC/Gal-BSA/DHA group (100 mg/kg) (*n* = 3). Then the corresponding nanoemulsions were given orally once a day for 7 d. After treatment, blood, heart, liver, spleen, lung and kidney were taken for follow-up tests. The blood of mice was added to a disposable vacuum EDTA tube and analyzed with an automatic blood cell analyzer (BC-5000vet, Mindray Biomedical, Shenzhen, China). At the same time, other tissues were fixed with 4% paraformaldehyde, paraffin-embedded sections, and histological H&E staining.

**Statistical analysis**

The results were expressed as the mean ± standard deviation (SD). Group comparisons were performed using one-way ANOVA. Depending on the outcome of the variance homogeneity test (e.g., Bartlett’s test), either ordinary one-way ANOVA (followed by Tukey’s *post hoc* test), Welch ANOVA (followed by the Games-Howell test), or Brown-Forsythe ANOVA was applied for multiple comparisons. Differences were considered statistically significant when *P* < 0.05. GraphPad Prism®9.5 was used for statistical analysis.

**References**

1. Mitchell C, Willenbring H. A reproducible and well-tolerated method for 2/3 partial hepatectomy in mice. *Nat Protoc* **2008**;3:1167-1170.
2. Xie G, Wang Y, Wang X, Zhao A, Chen T, Ni Y, Wong L, Zhang H, Zhang J, Liu C, Liu P, Jia W. Profiling of serum bile acids in a healthy Chinese population using UPLC-MS/MS. *J Proteome Res* **2015**;14:850-859.

**Figures**


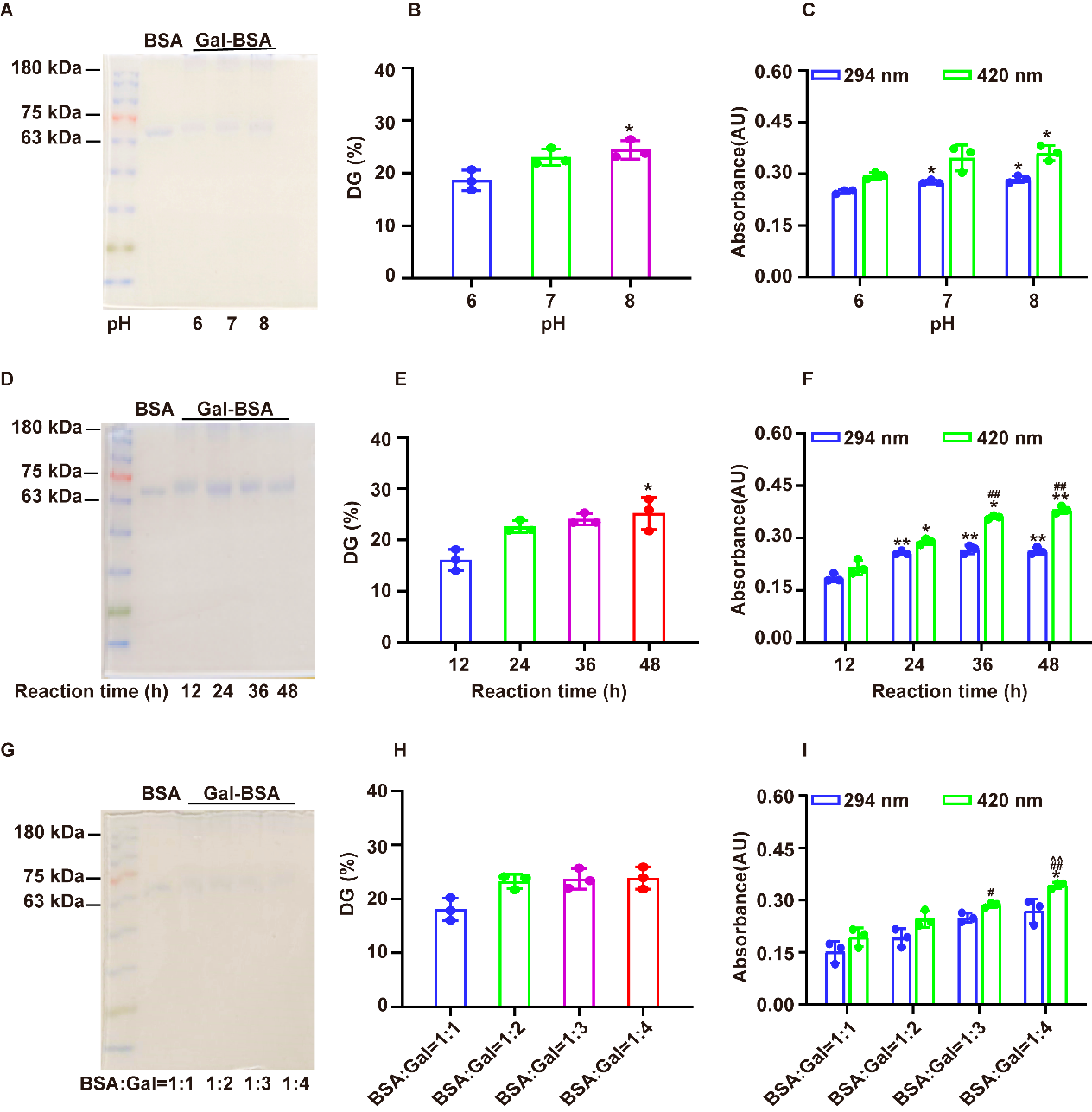


**Figure S1.** Preparation and optimization of Gal-BSA. (A) The SDS-PAGE, (B) DG, and (C) absorbance of Gal-BSA at different pHs. ^*^*P* < 0.05 compared with pH 6. (D) The SDS-PAGE, (E) DG, and (F) absorbance of Gal-BSA at different reaction times. ^*^*P* < 0.05 and ^**^*P* < 0.01 compared with 12 h. ^##^*P* < 0.01 compared with 24 h. (G) The SDS-PAGE, (H) DG, and (I) absorbance at 294 nm and 420 nm of Gal-BSA at different ratios. ^*^*P* < 0.05 compared with BSA:Gal = 1:1; ^##^*P* < 0.01 compared with BSA:Gal = 1:2; ^^^^*P* < 0.01 compared with BSA:Gal = 1:3. All data are presented as the mean ± SD (*n* = 3).


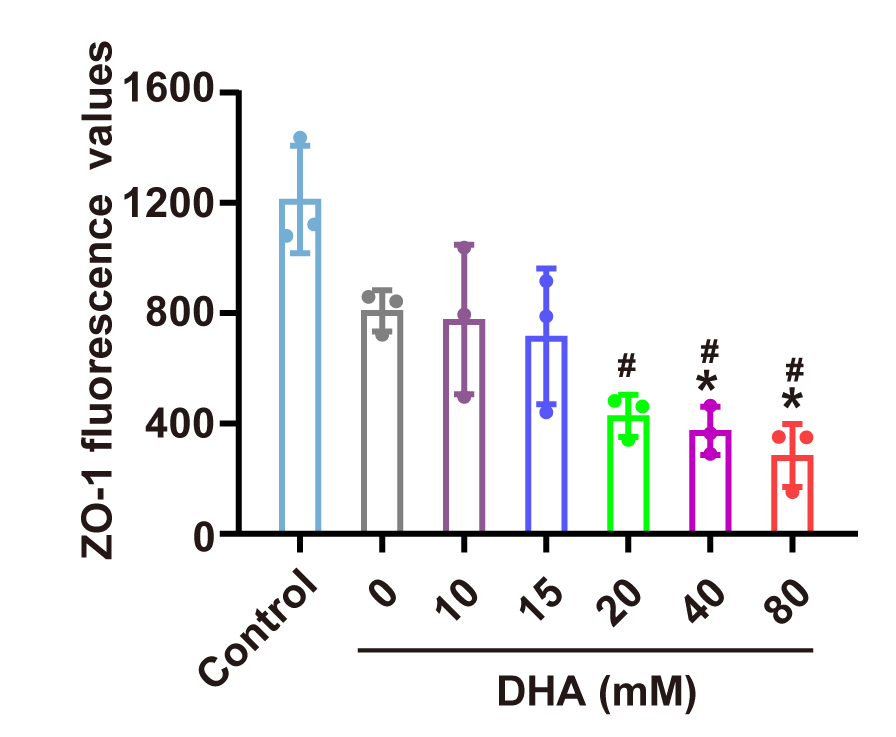


**Figure S2.** Fluorescence quantification of ZO-1 staining of SCHB@SPC/Gal-BSA/DHA at different DHA contents. ^*^*P* < 0.05 compared with control, ^#^*P* < 0.05 compared with 0 mM. All data are presented as the mean ± SD (*n* = 3).


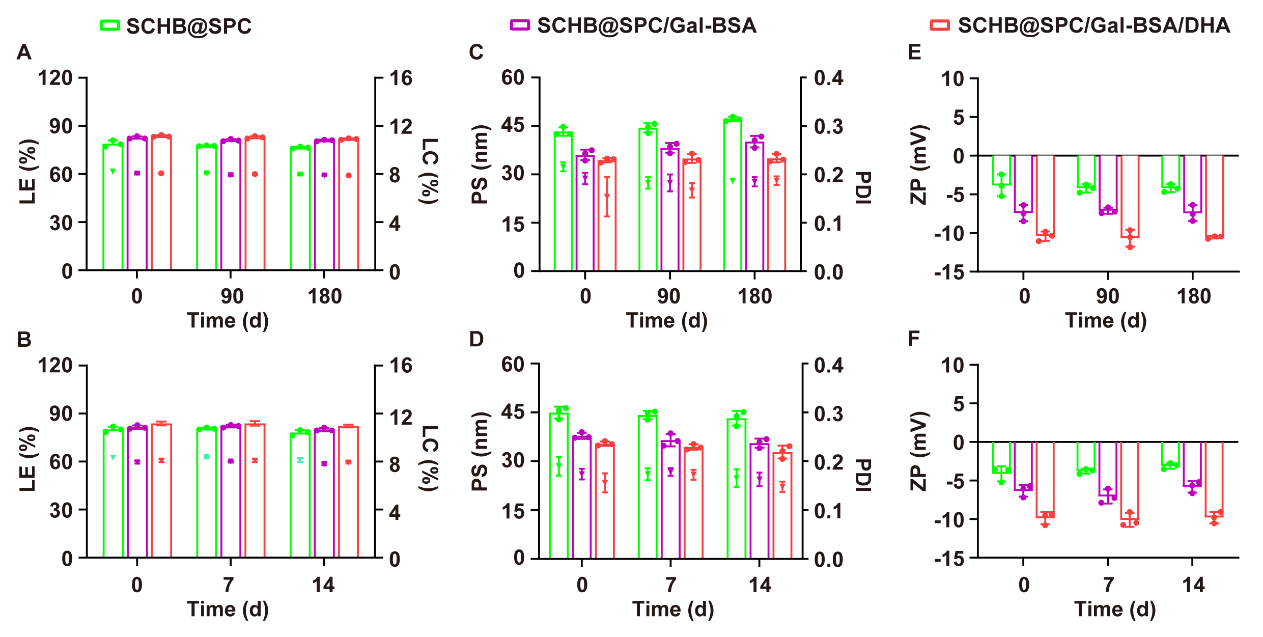


**Figure S3.** Changes in (**A** and **B**) LE and LC, (**C** and **D**) PS and PDI, and (**E** and **F**) ZP of SCHB nanoemulsions stored at 25℃ for 6 months and 40℃ for 2 weeks. All data are presented as the mean ± SD (*n* = 3).


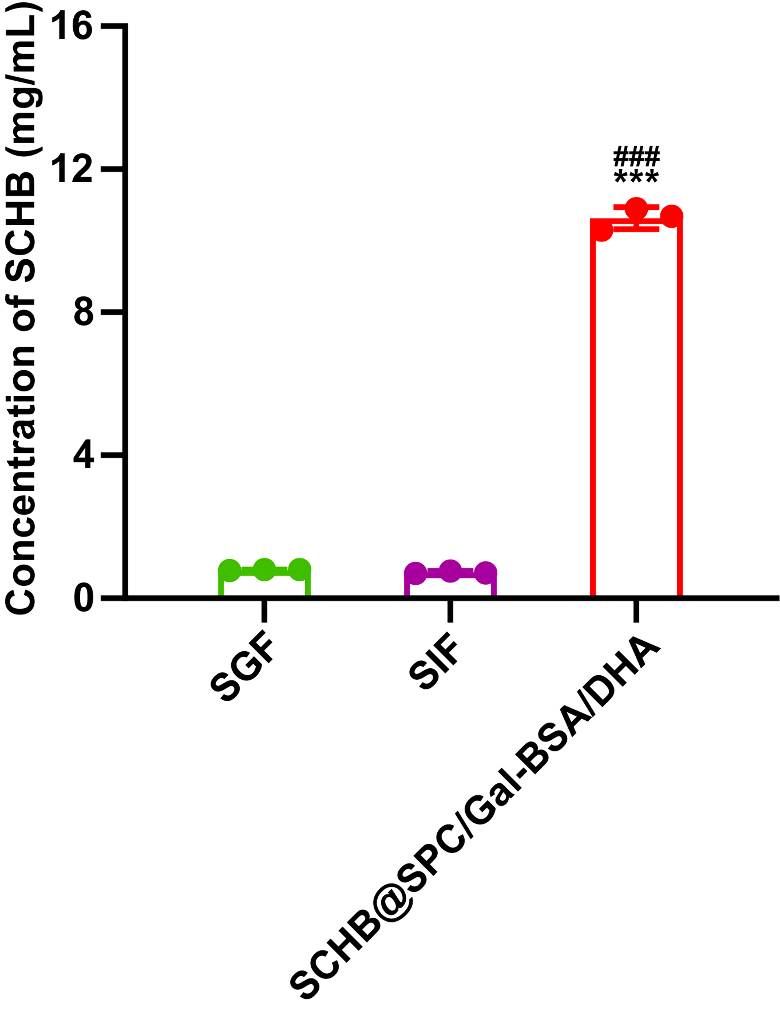


**Figure S4.** Determination of SCHB saturation solubility in SGF and SIF, ^***^*P* < 0.001 compared with SGF. ^###^*P* < 0.001 compared with SIF. All data are presented as the mean ± SD (*n* = 3).


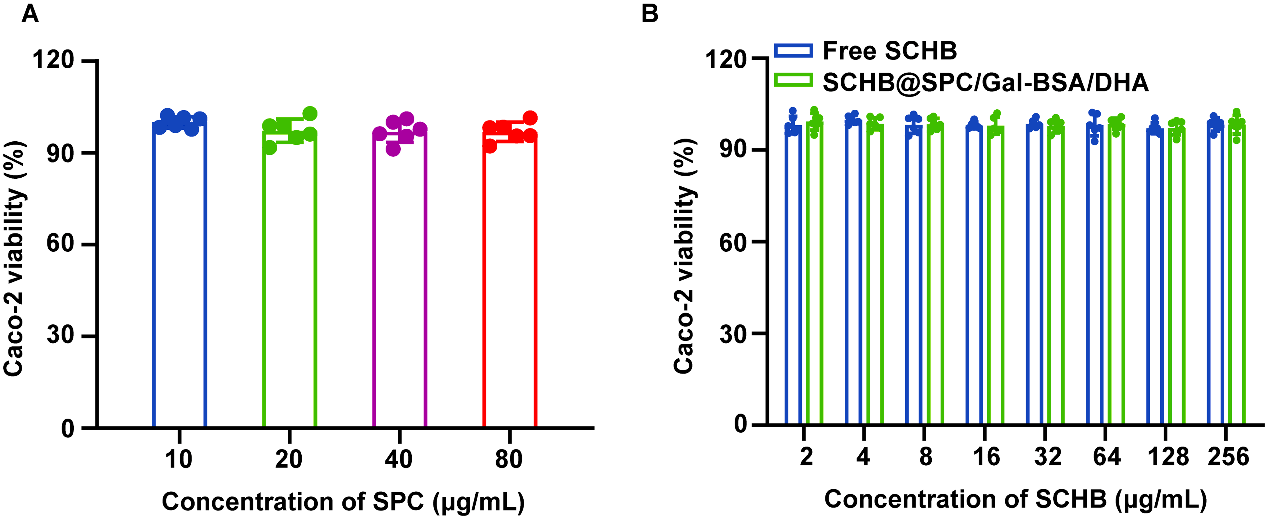


**Figure S5.** Safety evaluation of Caco-2 cells. (A) The cell viability of Caco-2 with different concentrations of SPC. (B) Determination of Caco-2 cell viability by free SCHB and SCHB@SPC/Gal-BSA/DHA at different concentrations. All data are presented as the mean ± SD (*n* = 6).


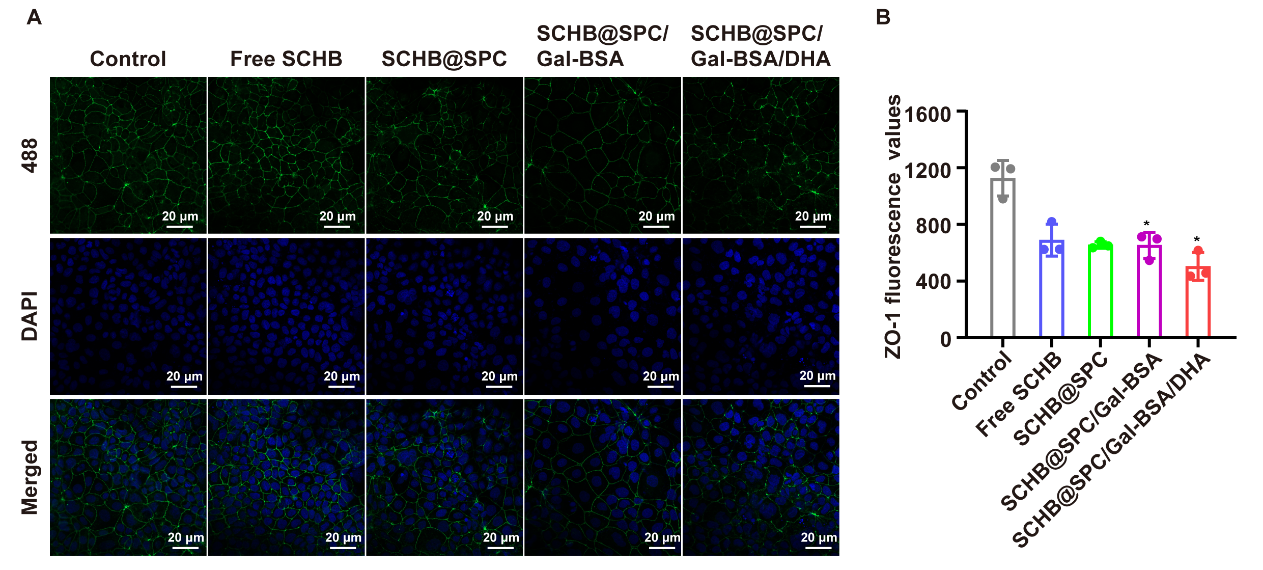


**Figure S6.** (A) ZO-1 staining and (B) fluorescence quantification of SCHB nanoemulsions. Scale bar 20 μm. ^*^*P* < 0.05 compared with control. All data are presented as the mean ± SD (*n* = 3).


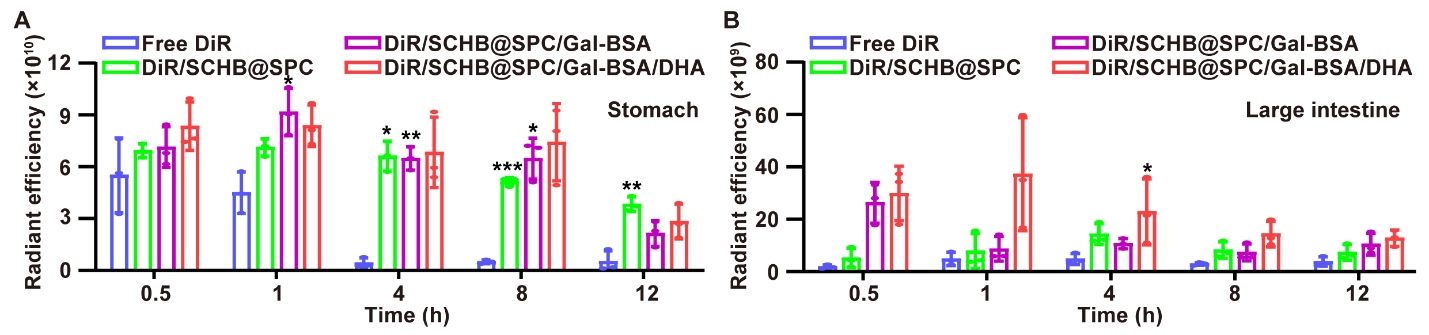


**Figure S7.** Fluorescent quantification of free DiR, DiR/SCHB@SPC, DiR/SCHB@SPC/Gal-BSA, and DiR/SCHB@SPC/Gal-BSA/DHA in the (A) stomach and (B) large intestine at various time points after oral administration. ^*^*P* < 0.05, ^**^*P* < 0.01 and ^***^*P* < 0.001 compared with free DiR. All data are presented as the mean ± SD (*n* = 3).

**
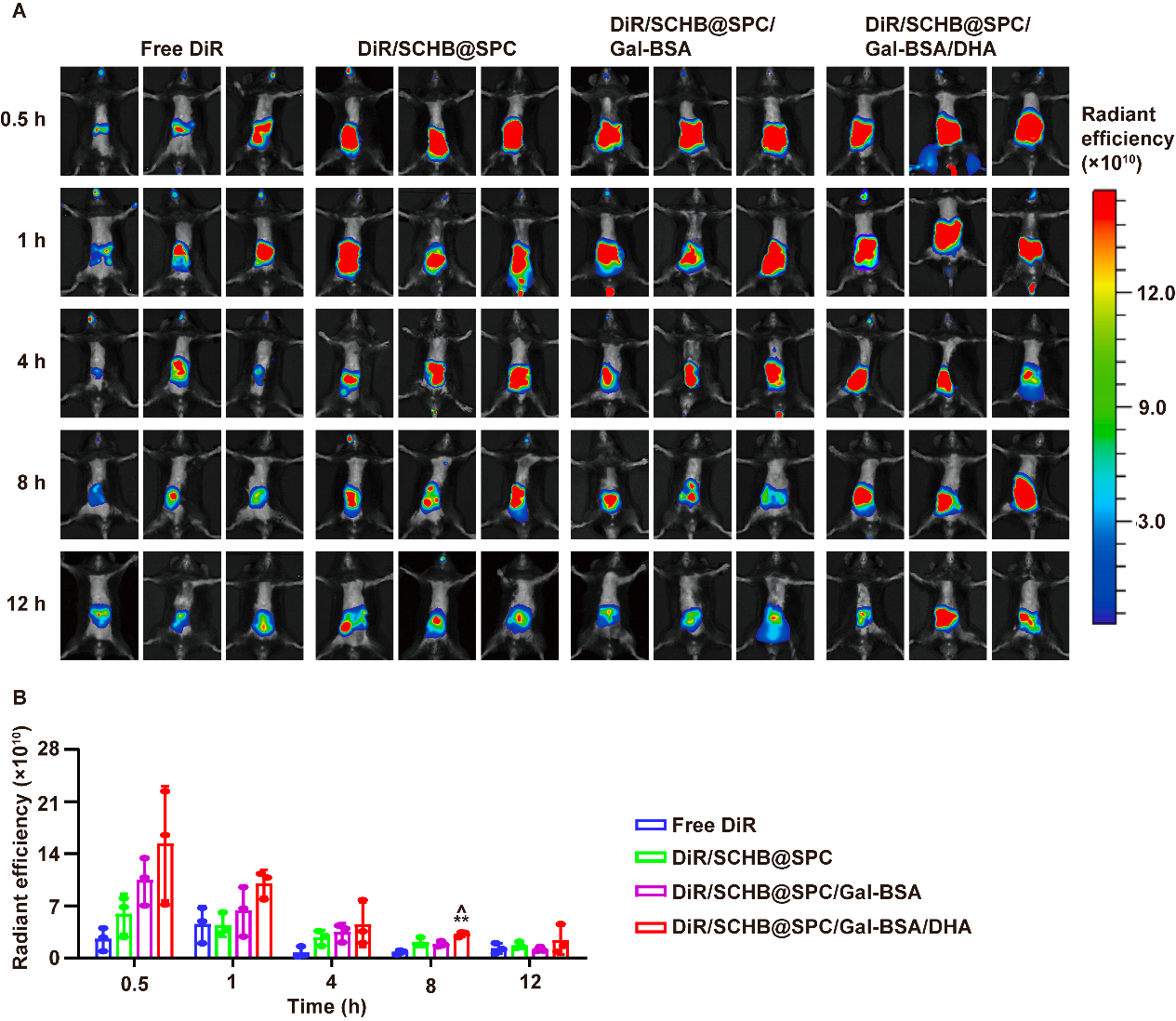
**

**Figure S8.** (A) Biological distribution and (B) fluorescence quantification of DiR nanoemulsions by IVIS. ^**^*P* < 0.01 compared with free DiR; ^^^*P* < 0.05 compared with DiR/SCHB@SPC/Gal-BSA. All data are presented as the mean ± SD (*n* = 3).


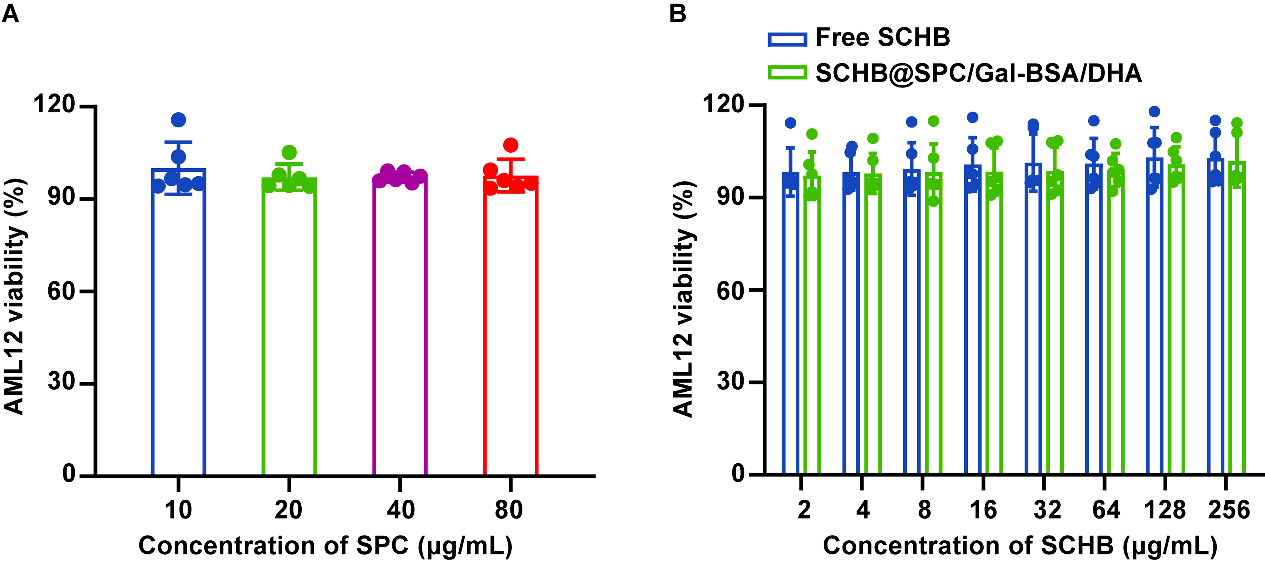


**Figure S9.** Safety evaluation of AML12 cells. (A) The cell viability of AML12 with different concentrations of SPC. (B) Determination of AML12 cell viability by free SCHB and SCHB@SPC/Gal-BSA/DHA at different concentrations. All data are presented as the mean ± SD (*n* = 6).


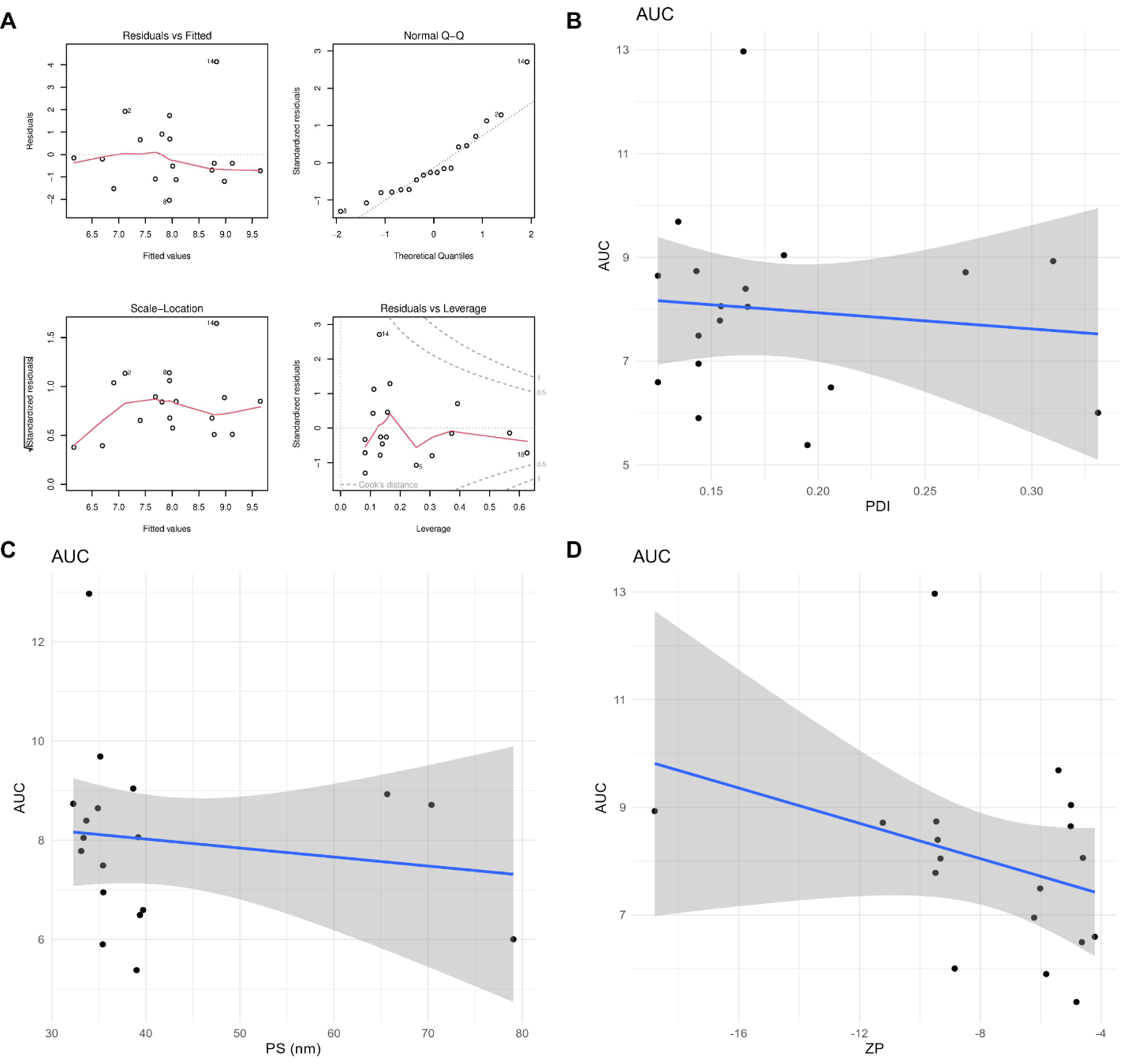


**Figure S10.** The correlation between nanoemulsion parameters and serum AUC.


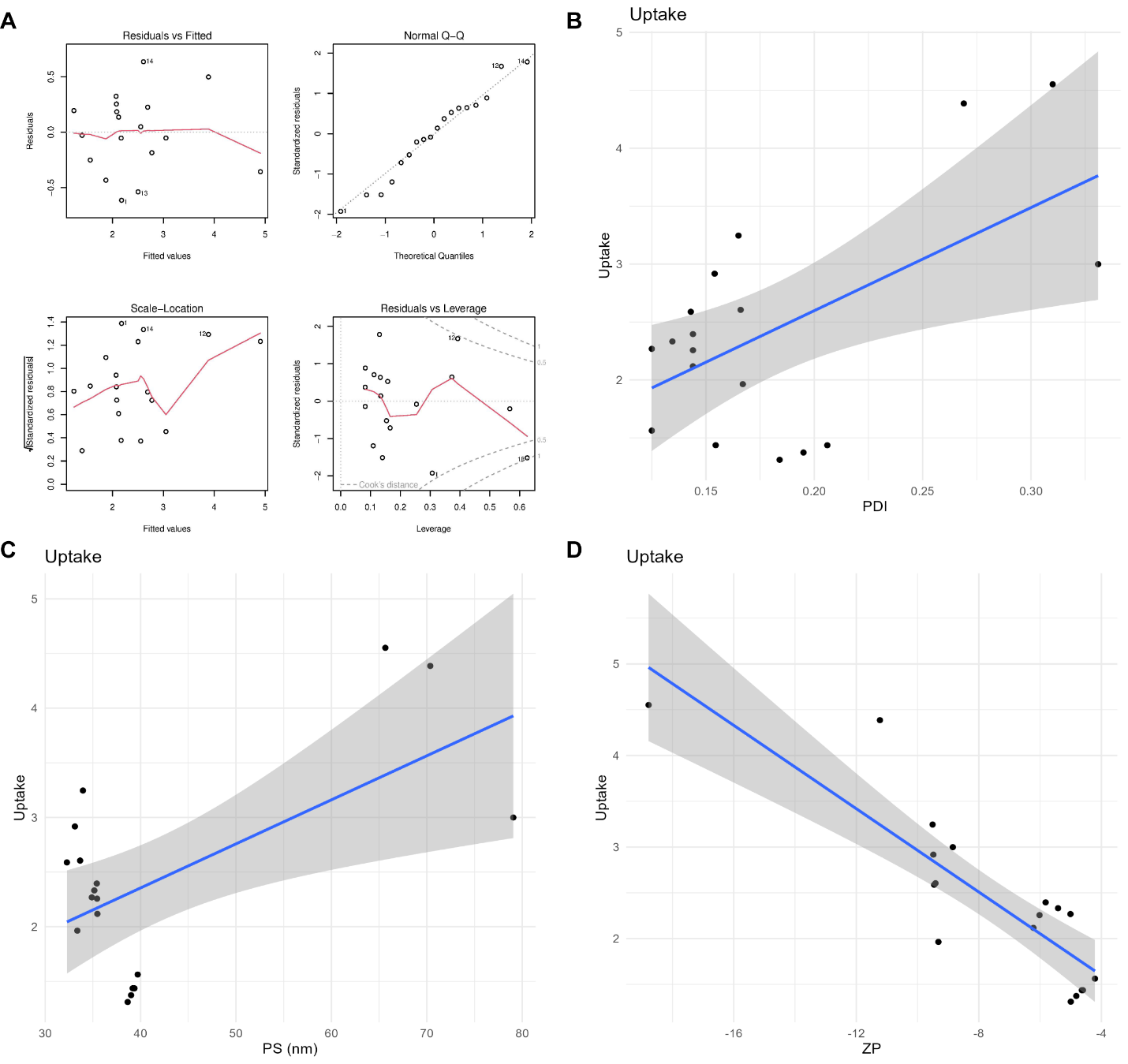


**Figure S11.** The correlation between nanoemulsion parameters and AML12 cells uptake.


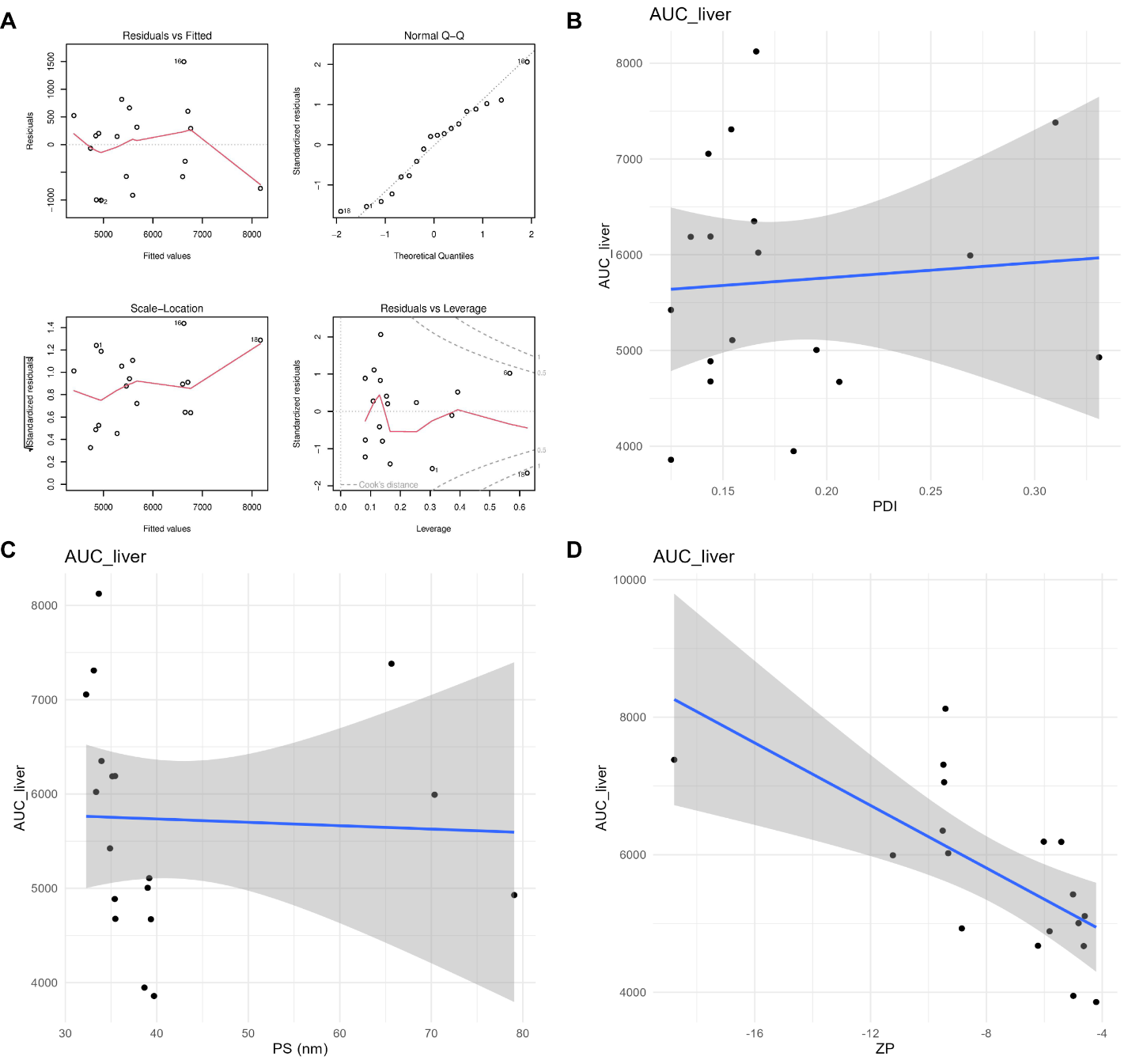


**Figure S12.** The correlation between nanoemulsion parameters and liver AUC.


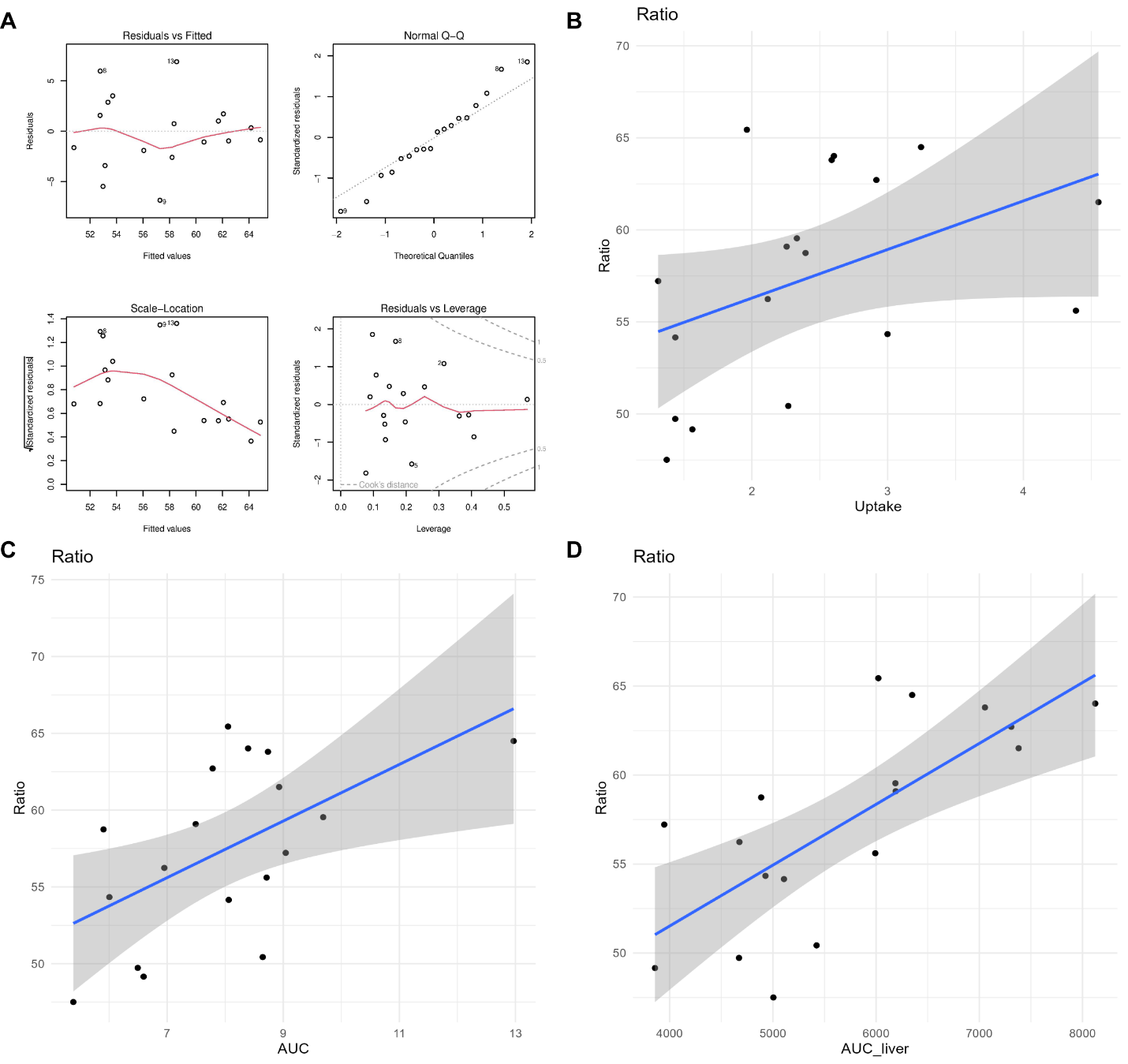


**Figure S13.** The correlation between pharmacodynamics parameters and liver regeneration.


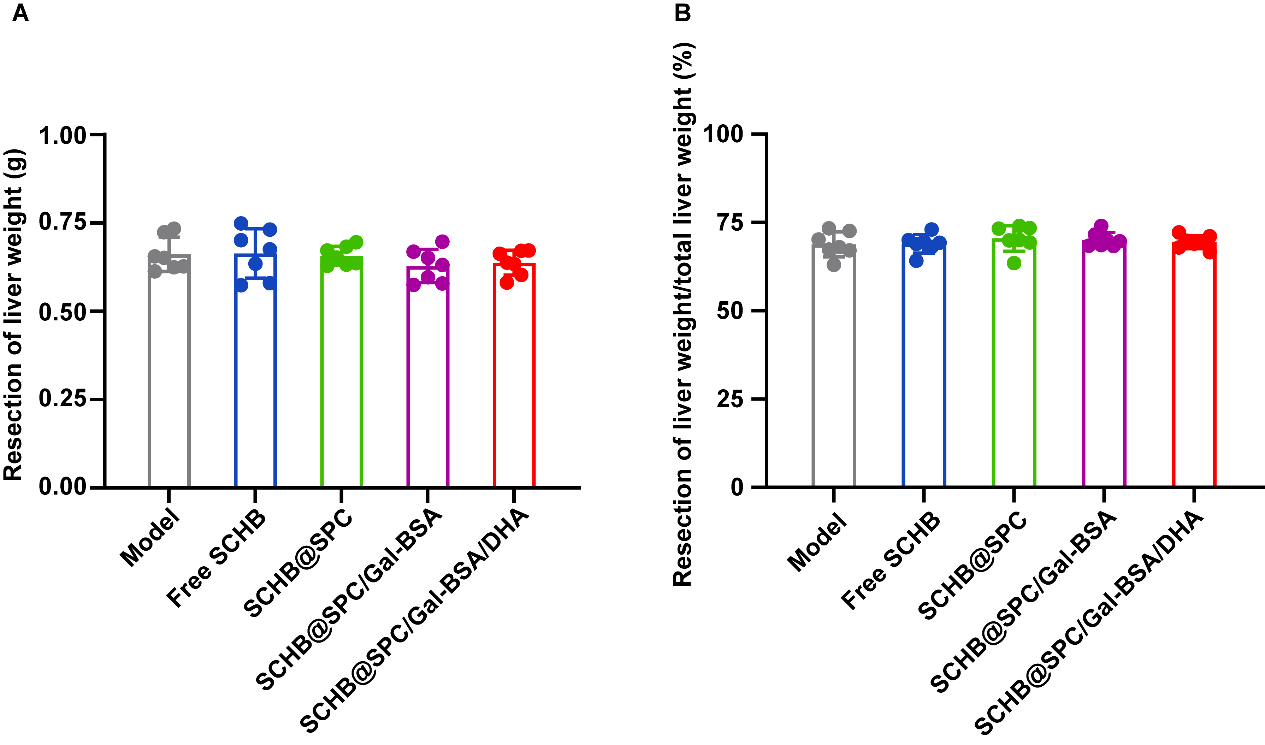


**Figure S****14.** The evaluation of the 70% PHx model. (A) The liver weight of resection in 70% PHx model. (B) Resection of liver weight/total liver weight. All data are presented as the mean ± SD (*n* = 7).


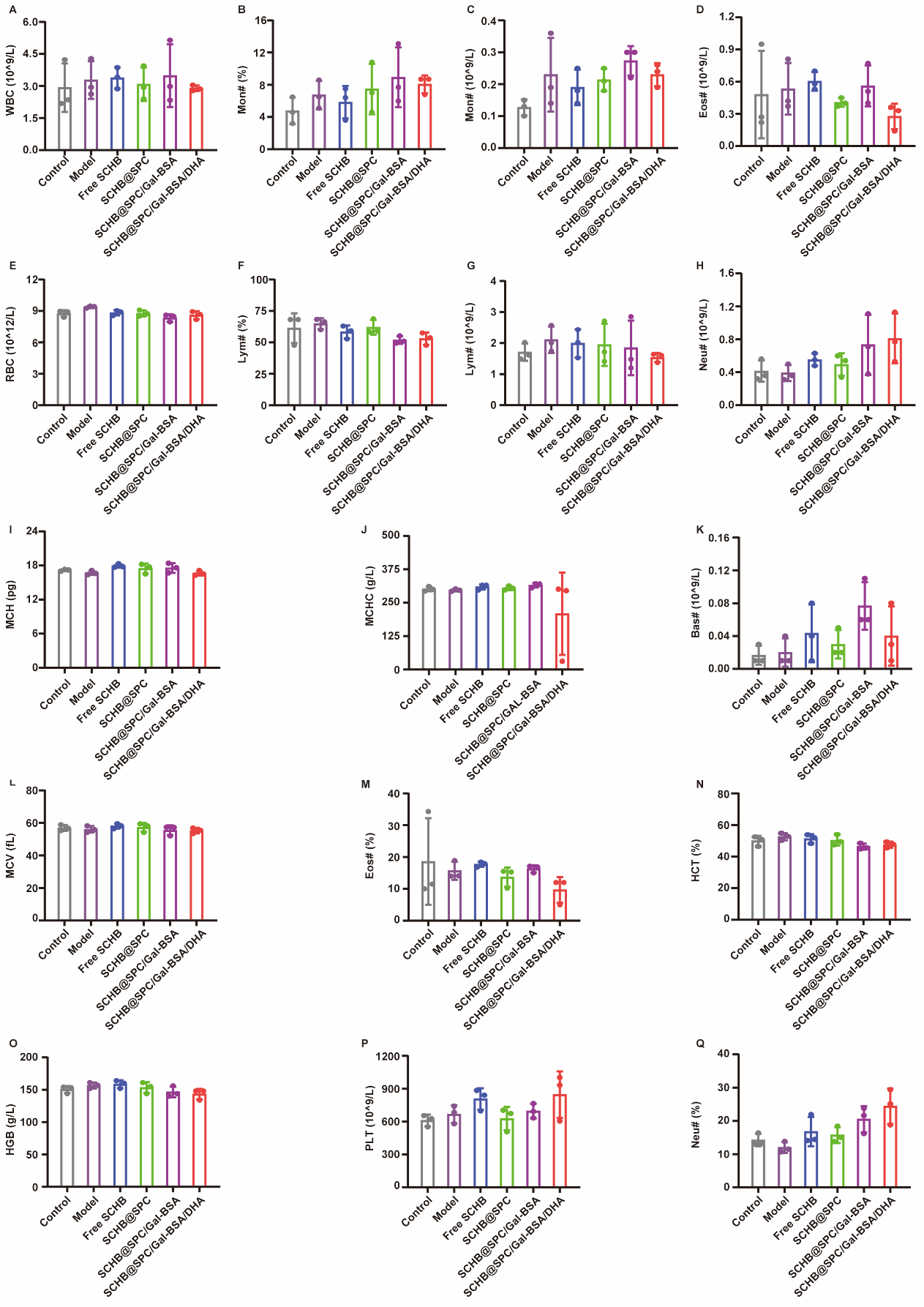


**Figure S15.** Complete blood count test after 7 d of oral SCHB nanoemulsions. (A) The white blood cell (WBC), (B) monocyte percentage (Mon%), (C) monocyte count (Mon#), (D) eosinophil count (Eos#), (E) red blood cell (RBC), (F) lymphocyte percentage (Lym%), (G) lymphocyte count (Lym#), (H) neutrophil count (Neu#), (I) mean corpuscular hemoglobin (MCH), (J) mean corpuscular hemoglobin concentration (MCHC), (K) basophil count (Bas#), (L) mean corpuscular volume (MCV), (M) eosinophil percentage (Eos#), (N) hematocrit (HCT), (O) hemoglobin (HGB), (P) platelet count (PLT), and (Q) neutrophil percentage (Neu#) in mice treated with SCHB nanoemulsions, respectively. All data are presented as the mean ± SD (*n* = 3).


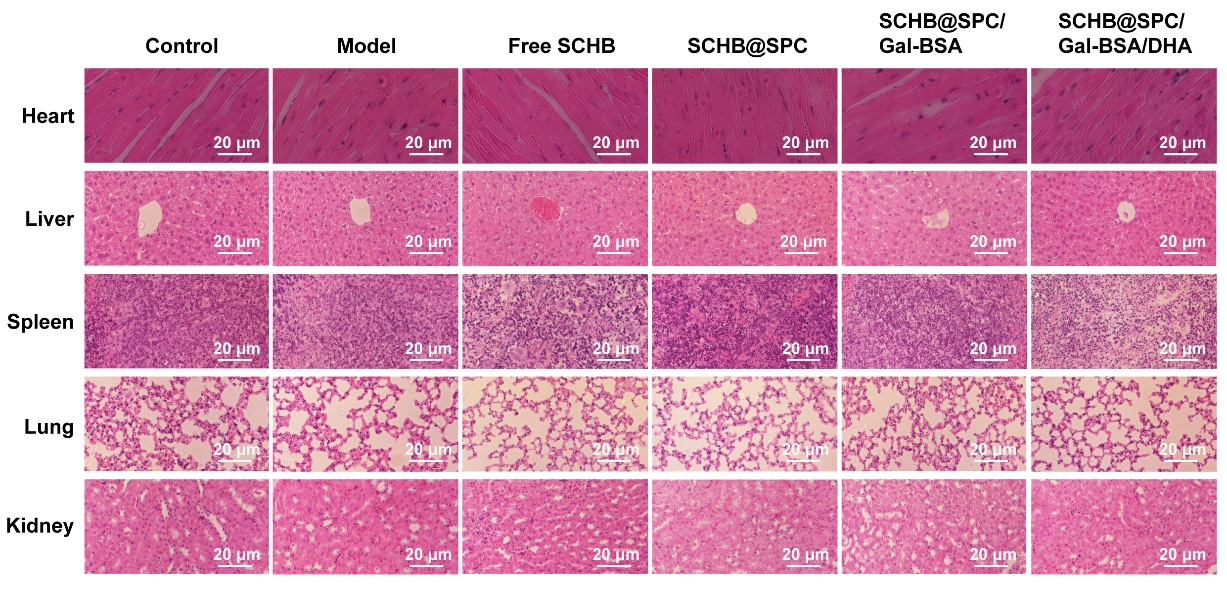


**Figure S16.** The H&E staining of the heart, liver, spleen, lung, and kidney in each group of safety evaluation (*n* = 3). Scale bar 20 μm.

**
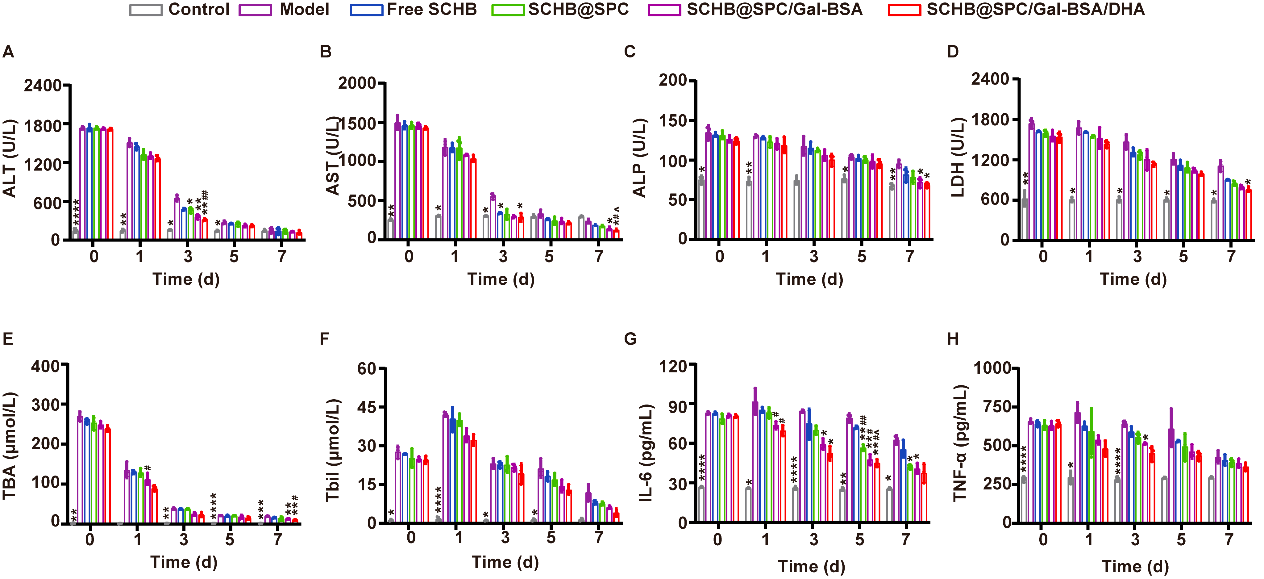
**

**Figure S17.** Levels of serum (A) ALT, (B) AST, (C) ALP, (D) LDH, (E) TBA, (F) Tbil, (G) IL-6 and (H) TNF-α in mice after 70% PHx. ^*^*P* < 0.05, ^**^*P* < 0.01, ^***^*P* < 0.001, and ^****^*P* < 0.0001 compared with model; ^#^*P* < 0.05 and ^##^*P* < 0.01 compared with free SCHB; ^^^*P* < 0.05 compared with SCHB@SPC. All data are presented as the mean ± SD (*n* = 3).

**
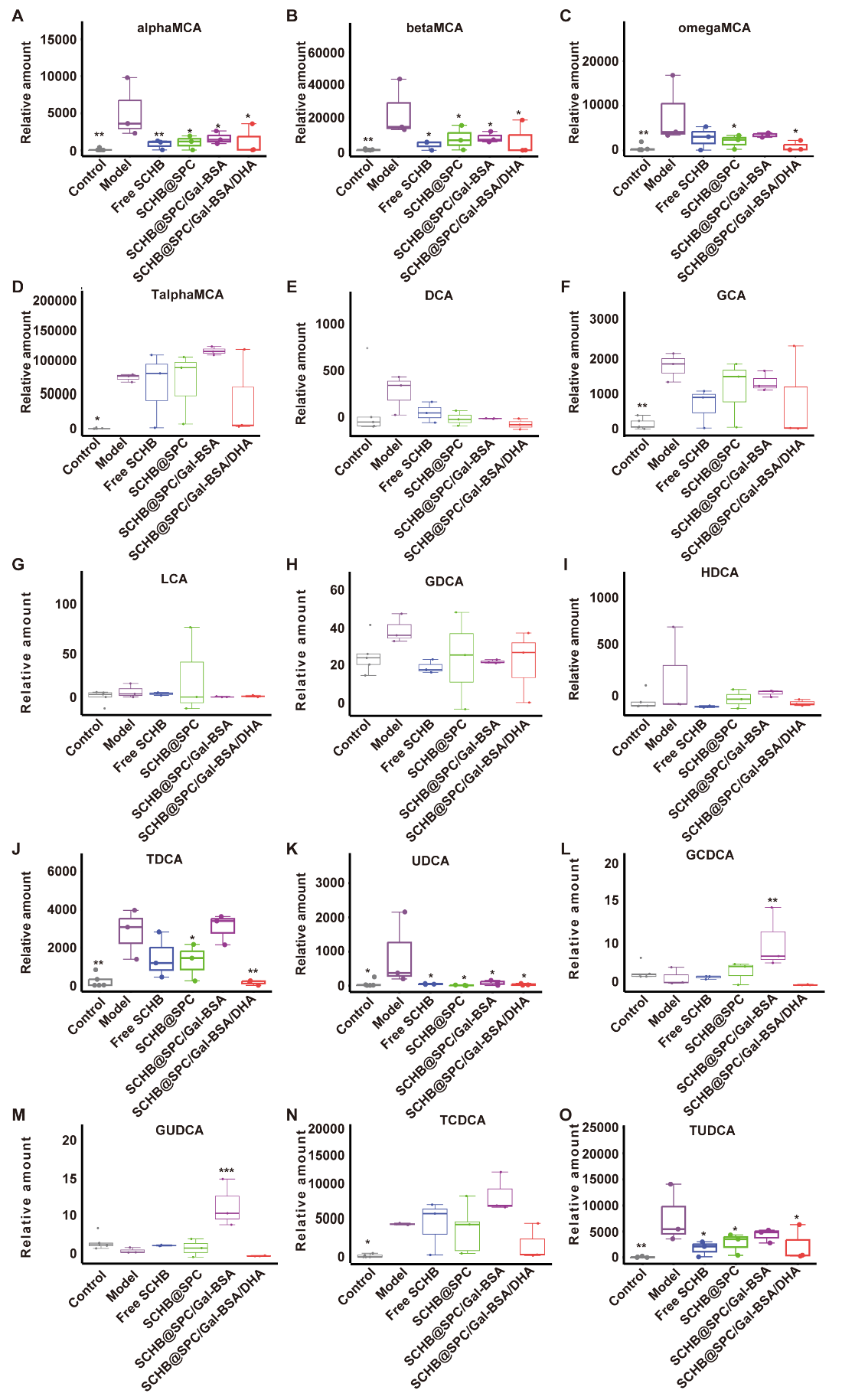
**

**Figure S18.** The relative amounts of (A) alphaMCA, (B) betaMCA, (C) omegaMCA, (D) TalphaMCA, (E) DCA, (F) GCA, (G) LCA, (H) GDCA, (I) HDCA, (J) TDCA, (K) UDCA, (L) GCDCA, (M) GUDCA, (N) TCDCA, and (O) TUDCA in the serum on 1 d after 70% PHx. ^*^*P* < 0.05, ^**^*P* < 0.01, ^***^*P* < 0.001, and ^****^*P* < 0.0001 compared with model. All data are presented as the mean ± SD (*n* = 3).

**
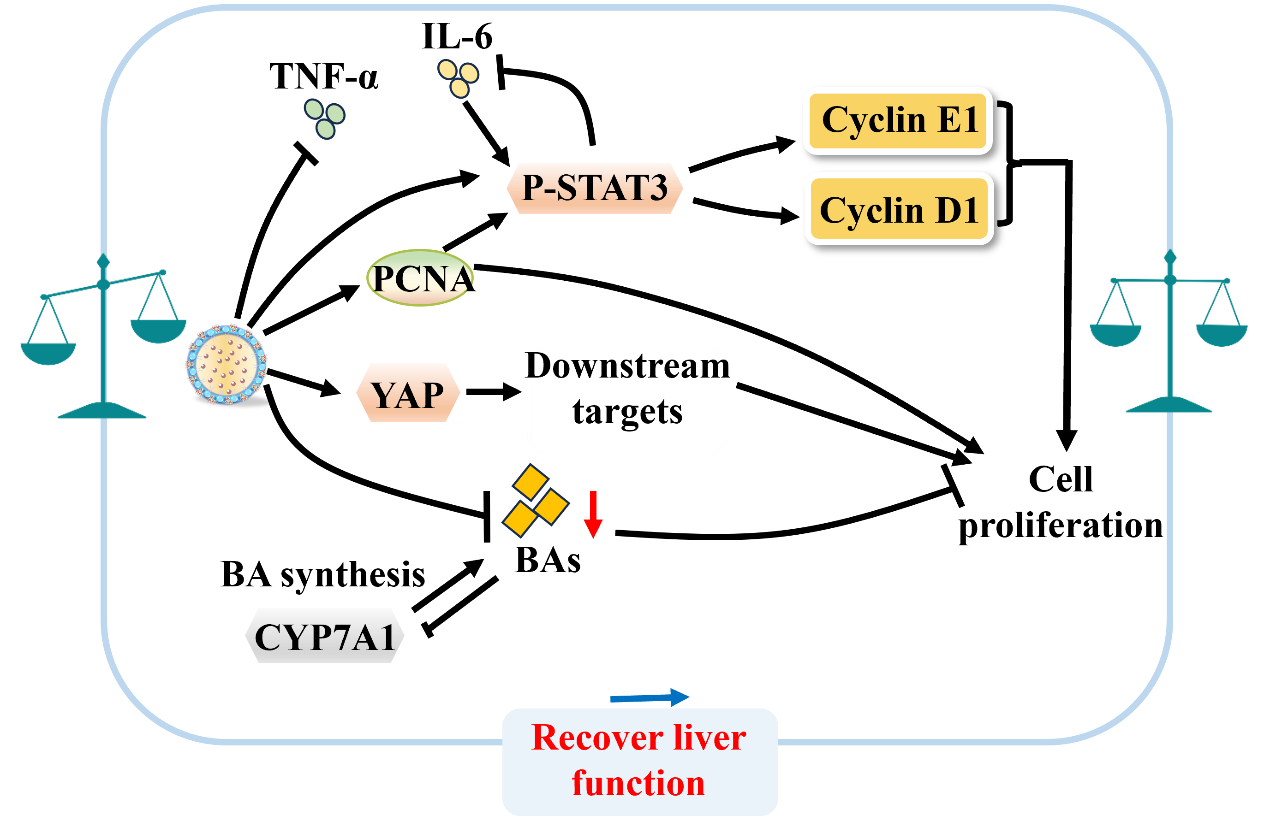
**

**Figure S19.** SCHB nanoemulsion mechanism diagram for promoting liver regeneration.


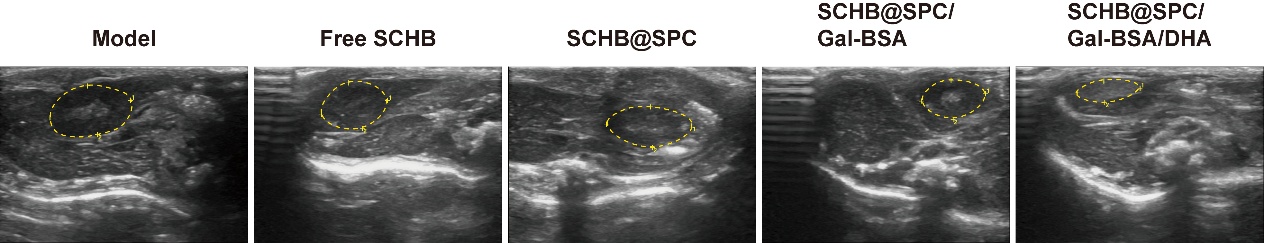


**Figure S20.** The USG of HCC mouse model (*n* = 3). The yellow dotted box shows liver tumors in HCC mice.


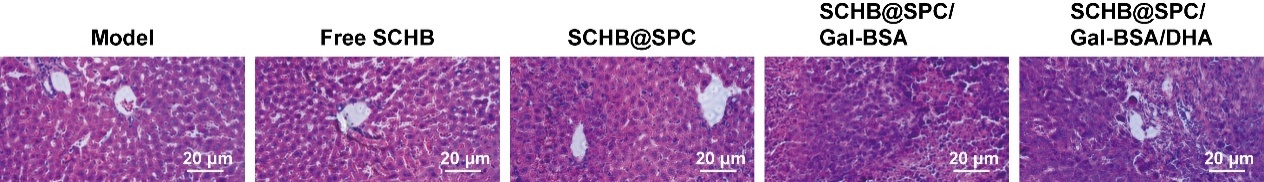


**Figure S21.** H&E staining of HCC mouse model (*n* = 3). Scale bar 20 μm.

**
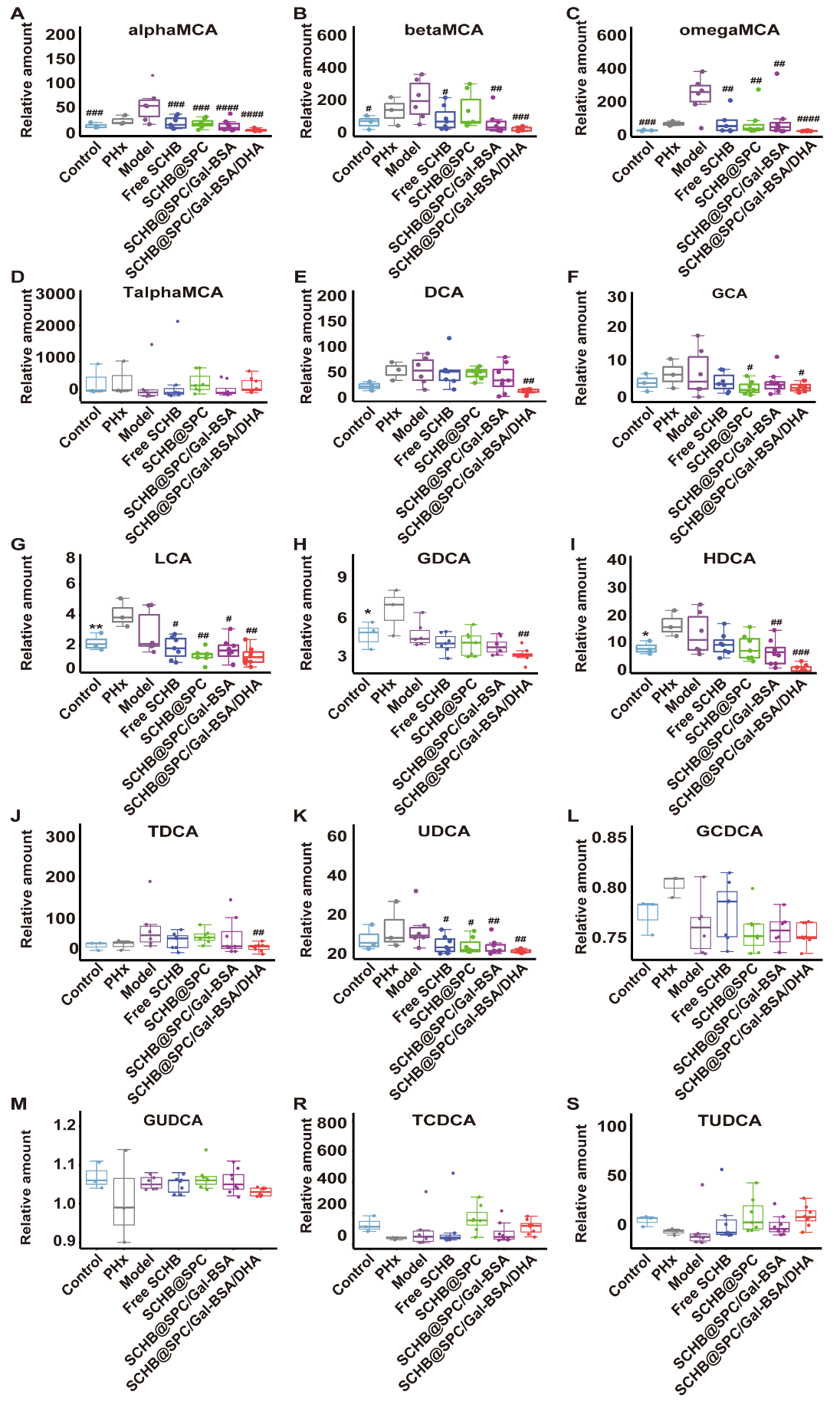
**

**Figure S22.** The relative amounts of (A) alphaMCA, (B) betaMCA, (C) omegaMCA, (D) TalphaMCA, (E) DCA, (F) GCA, (G) LCA, (H) GDCA, (I) HDCA, (J) TDCA, (K) UDCA, (L) GCDCA, (M) GUDCA, (N) TCDCA, and (O) TUDCA in the serum on 7 d after *in situ* PHx in HCC mice. ^*^*P* < 0.05 and ^**^*P* < 0.01 compared with PHx; ^#^*P* < 0.05, ^##^*P* < 0.01, ^###^*P* < 0.001, and ^####^*P* < 0.0001 compared with model. All data are presented as the mean ± SD (*n* = 8).

**Table**

**Table S1** The Geometric mean and confidence intervals of bioavailability and liver accumulation of intravenous injection free SCHB, oral administration free SCHB and SCHB nanoemulsions.

| **Parameters** | **Free SCHB (p.o.)** | **SCHB@SPC** | **SCHB@SPC/**  **Gal-BSA** | **SCHB@SPC/**  **Gal-BSA/DHA** |
| --- | --- | --- | --- | --- |
| Bioavailability (%) | 12.73 (10.68-15.18) | 19.56 (15.89-24.08)^**^ | 22.35 (18.58-26.88)^***^ | 25.81 (21.13-31.52)^****^ |
| Liver accumulation  (h*μg/mL) | 1971.85 (1744.07-2229.36) | 4557.99 (4001.74-5191.55)^****^ | 5524.98 (4856.3-6285.74)^****^ | 7005.75 (6250.65-7852.07)^****^ |

^*^*P* < 0.05, ^**^*P* < 0.01, ^***^*P* < 0.001, and ^****^*P* < 0.0001 compared with free SCHB (i.g.).
